# Supplementary material for: Effect of patient-delivered household contact tracing and prevention for tuberculosis: A household cluster-randomised trial in Malawi
Source: PLoS One. 2022 Sep 8;17(9):e0269219. doi: 10.1371/journal.pone.0269219 (PMC9455850; doi:10.1371/journal.pone.0269219)
Supplement: S1 File — (DOCX) [file pone.0269219.s003.docx]

**Providing sustainable household contact tracing and screening for Tuberculosis Patients and Families: a Cluster-randomised trial in Blantyre, Malawi (PACTS trial).**

**Grant Holders: Dr Elizabeth Corbett and Dr Geoffrey Chipungu**

**Principal Investigator:** Kruger Kaswaswa

**Host Institutes:** University of Malawi, College of Medicine, Blantyre, and University

of Tromso (Norway)

**Supervisors:**

**MLW and College of Medicine and University of Tromso**

Dr Elizabeth Corbett (Reader) - **MLW and LSHTM**

Dr Bagrey Ngwira - **College of Medicine**

Dr Henry Mwandumba - **MLW**

**Co investigators:**

Dr James Mpunga **- NTP Manager, Ministry of Health**

Dr Neil Kennedy **- Department of Paediatrics and Child Health**

Dr Naor Bar_Zeev **- Department of Paediatrics and Child Health**

Dr Bernadetta O`Hare **-Department of Paediatrics and Child Health**

**Collaborators:**

Dr Theresa Allain **- Department of Medicine**

Dr Andrew Gonani -**Director Queens Elizabeth Central Hospital**

Dr Felix Salaniponi -**Share World Open University**

Dr Owen Malema -**District Health Officer Blantyre**

Mr Nauko Gama -**TB Officer DHO Blantyre**

Mrs Nyembezi Chinkhombe **-TB Officer QECH**

Signature: Date:

**Table of contents**

[Abbreviations 6](#_Toc332191063)

[1 Summary 7](#_Toc332191064)

[1.1 Introduction 7](#_Toc332191065)

[1.2 Trial design/concept 7](#_Toc332191066)

[1.3 Study Aims 8](#_Toc332191067)

[1.4 Methodology 8](#_Toc332191068)

[1.4.1 Study population 8](#_Toc332191069)

[1.4.2 Intervention 8](#_Toc332191070)

[1.4.3 Procedures in all participating household. 9](#_Toc332191071)

[1.5 Outcomes 9](#_Toc332191072)

[1.5.1 Primary outcome measures are: 9](#_Toc332191073)

[1.5.2 Secondary outcome measures 9](#_Toc332191074)

[1.6 Other planned outcome measures are: 10](#_Toc332191075)

[1.7 Study time line 10](#_Toc332191076)

[1.8 Intended use of results 10](#_Toc332191077)

[2 Background 11](#_Toc332191078)

[2.1 Household contact tracing for TB case detection 12](#_Toc332191079)

[2.2 Contact tracing and Isoniazid Preventive Therapy (IPT) 13](#_Toc332191080)

[2.3 Barriers to TB diagnosis in Malawi. 14](#_Toc332191081)

[2.4 Household contact policy and practice in Malawi 14](#_Toc332191082)

[2.5 TB infection and disease in children 15](#_Toc332191083)

[3 Study Rationale 16](#_Toc332191084)

[4 Research Question 16](#_Toc332191086)

[5 Research Aims and Objectives 16](#_Toc332191087)

[6 Specific Objectives 16](#_Toc332191088)

[7 Methodology 17](#_Toc332191089)

[*7.1* Trial Design 17](#_Toc332191090)

[7.2 Flow diagram 17](#_Toc332191091)

[7.3 Setting and Study population 18](#_Toc332191092)

[7.4 Intervention- 18](#_Toc332191093)

[7.5 Standard of Care Arm: 19](#_Toc332191094)

[7.6 Sample size 19](#_Toc332191095)

[7.7 Randomization 19](#_Toc332191096)

[7.8 Cohort Follow up and 3 month home visit 20](#_Toc332191097)

[8 Outcome evaluation 21](#_Toc332191098)

[8.1 Capture of trial outcomes 21](#_Toc332191099)

[8.2 Active TB cases 21](#_Toc332191100)

[8.3 IPT initiation and completion 21](#_Toc332191101)

[8.4 Data capture tools for active TB cases, and IPT initiation and completion 22](#_Toc332191102)

[8.5 Number of cluster residents registering for TB household screening care 22](#_Toc332191103)

[9 Data management and quality assurance 23](#_Toc332191104)

[9.1 TB screening in the households 23](#_Toc332191105)

[9.2 Data collection 23](#_Toc332191106)

[9.3 Data management 23](#_Toc332191107)

[9.3.1 Data collection tools, case record forms (CRF) 24](#_Toc332191108)

[9.3.2 Data security 24](#_Toc332191109)

[10 Statistical consideration 24](#_Toc332191110)

[10.1 Statistical analysis 24](#_Toc332191111)

[11 Ethical consideration 25](#_Toc332191112)

[11.1 Participant information and consent forms 25](#_Toc332191113)

[11.2 Ethics Committees providing review and approval 25](#_Toc332191114)

[12 Trial governance 26](#_Toc332191115)

[12.1 Trial Steering Committee 26](#_Toc332191116)

[12.2 Trial registration 26](#_Toc332191117)

[12.3 Data Safety Monitoring Board (DSMB) 26](#_Toc332191118)

[12.4 Good Clinical Practice 26](#_Toc332191119)

[12.5 Monitoring and audit 26](#_Toc332191120)

[13 Dissemination 27](#_Toc332191121)

[13.1 Policy for sharing data 27](#_Toc332191122)

[13.2 Dissemination to communities 27](#_Toc332191123)

[14 Possible constraints 27](#_Toc332191124)

[15 Budget 27](#_Toc332191125)

[15.1 Budget Justifications 27](#_Toc332191126)

[15.2 Study start up costs. 28](#_Toc332191127)

[15.3 Salaries and wages 28](#_Toc332191128)

[15.4 Other study support direct costs 28](#_Toc332191129)

[15.5 Supplies and materials (Laboratory). 28](#_Toc332191130)

[15.6 Travel 29](#_Toc332191131)

[15.7 Dissemination and publications 29](#_Toc332191132)

[16 Training Provided for 31](#_Toc332191133)

[17 Time frame 32](#_Toc332191134)

[18 References 33](#_Toc332191135)

[19 Annexes 37](#_Toc332191136)

[19.1 Study suggested pathway for children 37](#_Toc332191137)

[19.2 Study definitions 38](#_Toc332191138)

[19.3 IPT provision 39](#_Toc332191139)

[19.3.1 Isoniazid preventive therapy 39](#_Toc332191140)

[19.3.2 Expected number of patients 39](#_Toc332191141)

[19.3.3 Inclusion criteria for IPT initiation (MOH) 39](#_Toc332191142)

[19.3.4 Exclusion criteria (absolute contra-indications): - 39](#_Toc332191143)

[19.3.5 Dose and duration of IPT 39](#_Toc332191144)

[19.3.6 Safety and monitoring during IPT 40](#_Toc332191145)

[19.3.7 Hepatitis 40](#_Toc332191146)

[19.3.8 Peripheral neuropathy 40](#_Toc332191147)

[19.3.9 Treatment of established isoniazid neuropathy 40](#_Toc332191148)

[19.3.10 Dispensing 41](#_Toc332191149)

[19.3.11 Adherence 41](#_Toc332191150)

[19.3.12 Recording and reporting adverse events 41](#_Toc332191151)

[19.4 TB case notification in Blantyre 2010 42](#_Toc332191152)

[19.5 Sample size calculations for primary outcome 1 42](#_Toc332191153)

[19.6 Training 42](#_Toc332191154)

[19.7 Household Sputum Collection Procedures: 43](#_Toc332191155)

[19.8 Ethical Approval and consent Forms 44](#_Toc332191156)

[19.9 Information sheet and Consent Forms: At 3 month household screening. 49](#_Toc332191157)

[19.10 Uthenga ndi Chilolezo: Kulemba ndi kulowetsa anthu odwala matenda a TB mukafufukuyi. (At recruitment -Chichewa version) 54](#_Toc332191158)

[19.11 Uthenga ndi Chilolezo: Kufufuku wofufuza za matenda a TB pa khomo patatha miyezi itatu (At 3 month household screening -Chichewa version). 59](#_Toc332191159)

# Abbreviations

CPT - Cotrimoxazole Preventive Therapy

AIDS - Acquired Immunodeficiency Syndrome

ART - Anti Retroviral Therapy

DHO - District Health Office

DOT - Direct Observed Treatment

DOTS - Direct Observed Treatment, Short course

DTO - District Tuberculosis Office

EPTB - Extra Pulmonary Tuberculosis

GOM - Government of Malawi

HIV - Human Immunodeficiency Virus

IPT - Isoniazid Preventive Therapy

MNTP - Malawi National Tuberculosis Control Programme

MOH - Ministry of health

NTP - National Tuberculosis Control Programme

PTB - Pulmonary Tuberculosis

TB - Tuberculosis

WHO - World Health Organization

ISRCTN - International Registered Clinical Trial Number

MRC - Medical Research Council UK

MLW - Malawi Liverpool Welcome Trust

LSHTM - London school of Hygiene and Tropical medicine

HNTI - Helse Nord TB initiative

PACTS - Providing Sustainable household contact tracing and screening in TB patients

# Summary

## Introduction

Household contact tracing with screening of all contacts of smear positive Mycobacterium *tuberculosis* (MTB) patients is national and international policy, aiming to reduce further transmission of TB. Household contact tracing and screening provides “active” early detection of TB cases that complements the traditional approach of “passive case detection” in Malawi. Active case-finding is considerably more effective than passive case-finding alone, but also more expensive to implement than offering services passively. Contact screening identifies candidates for both TB treatment and preventive therapy with isoniazid. In Malawi, household contact screening is mainly facility based, with health workers advising index cases to bring their contacts for symptom screening, followed by clinical investigations (sputum smear microscopy, chest radiography) if symptomatic. However this service has been poorly implemented and utilized, as evidenced by a large gap between the expected number of contacts eligible for screening and IPT and the numbers brought to the national programme. We hypothesise that this relates to the high investment of transport costs and time that TB patients and their families need to commit in order to attend and complete the screening process.

## Trial design/concept

This will be a cluster randomized trial of approximately 1712 individual household contacts of 428 index TB patients (assuming that each household has an average of four contacts). The unit of randomization will be the index case. Index patients will be randomly assigned to either *standard of care* or *intervention* arm. The intervention will consist of the following components: -

1. Initiating contact screening at home by the patient themselves, with the index patient then also responsible for: -
   1. Triaging household members into contacts who need TB investigation, or are eligible for IPT, and those where no further action is indicated
   2. Collecting and bringing sputum for examination from symptomatic household members who are otherwise well
   3. Initiating other actions as indicated, aided by “contact referral cards” that can be used to directly access paediatric services for under 5 year old family members with symptoms of TB
2. The index patient can then bring all children eligible for IPT with them to their next routine appointment (TB drugs are collected weekly from primary care clinics), where IPT can be initiated. If the index patient is not the parent/guardian, then the child(ren) will have to be accompanied by their parent/guardian
3. Thereafter the index patient, or parent of the children (if not the index patient) can assume responsibility for collecting isoniazid and carrying out monthly symptom screening for isoniazid side-effects on behalf of all family members, thereby minimizing the need for travel

Participants will have brief training on symptom screening and household triage at the time of registration and written informed consent. They will be provided with specimen containers and illustrated instructions to enable sputum collection in adults and older children, and “contact referral cards” to take home with them. Further training on symptom screening and recording administration of IPT (similar to TB DOT cards) will be provided to the index patient/parent if IPT is subsequently started.

## Study Aims

The broad objective of this trial is to pilot, implement and rigorously assess the effectiveness of a home- based TB screening (PACTS) strategy delivered to the households of TB patients in urban Blantyre, aiming to identify a potentially sustainable model that could improve uptake of TB cases with all components of the current contact screening policy of NTP Malawi.

**Specific Objectives**

1. To investigate the cumulative incidence of timely (within 3 months) diagnosis and treatment of TB in household contacts of adult index TB patients recruited from QECH, using a cluster randomized trial design to compare the PACTS intervention against standard of care (SOC)
2. To investigate the uptake of IPT by 3 months in under 5 year old child contacts of adult index TB patients recruited from QECH, comparing results between the two trial arms.
3. To investigate the proportion of child contacts in the two trial arms who both initiate *and* complete a 6 month course of IPT
4. To investigate the prevalence of undiagnosed TB by TB screening at a home visit conducted 3 months after the diagnosis of the index case

For objectives 1 to 3, initiation of TB treatment and IPT will be considered up to the time of (but not including) a planned 3 month household visit.

## Methodology

### Study population

Household contacts of new pulmonary TB patients registering for TB treatment in QECH will be defined as a cluster for study purposes. A total of 428 index cases will be recruited; giving a population of ~ 1712 adult and child contacts on the assumption that mean household size is 5 (giving 4 contacts per index patient).

### Intervention

Consenting TB patients who are randomized to the intervention arm will receive:

- A brief information leaflet
- A TB symptom checklist for adults and children in Chichewa
- A household screening card for self-completion that allows the index patient to document the name and age of all household members, presence or absent of TB symptoms, eligibility for IPT and next-steps (no further action / sputum microscopy / primary care visit with index for IPT initiation / QECH paediatric clinic)
- Sputum containers and bags
- A “contact referral card” for direct referral of children under the age of less than 5 years who have TB symptoms to the paediatric TB clinic in QECH

If IPT is started then the index patient or the parent/guardian of the child(ren) will be trained in use of an IPT Directly Observed Treatment card for recording treatment compliance, and in recognition of and screening for IPT side-effects, using materials adapted from the Practical Approach to Lung Health (PAL)(1). A contact number and the name of a focal person at the prescribing facility will be provided in case of any queries.

The index patient will be able to collect monthly IPT supplies with their own TB drugs without the child having to attend the facility. The index patient will have to show the IPT DOT treatment card and any remaining isoniazid tablets/syrup.

In the event that the Index patient stops attending (e.g. default, hospitalization, death or transfer out) then an alternative family member or guardian can assume responsibility for continuing IPT on completion of brief training.

### Procedures in all participating household.

On recruitment, the name, age, gender and TB status (TB treated or not) of all contacts will be recorded in MOH contact registers. Subsequently, MOH IPT registers will be used to capture IPT initiation and completion in both trial arms. Index cases in both trial arms will be provided with health education regarding side effects of IPT and benefits of completing IPT.

A home visit at 3 months will be carried out in all participating households, in order to screen for undiagnosed TB and IPT eligibility in household contacts and to confirm the primary outcomes (TB treatment started since the diagnosis of the index case). The 3 month household visit will also be used to identify all children initiated on IPT (second primary outcome).This will provide the cohort on which the secondary outcome analysis will be based

## Outcomes

### Primary outcome measures are:

- The cumulative incidence of all active TB in adults and child contacts three months after diagnosis of index cases.
- Proportion of household contacts less than 5 years old that start IPT within three months of the diagnosis of index cases (before the household screening at 3 months).

### Secondary outcome measures

- Proportion of all household contacts under 5 years old who complete a 6-month course of IPT within 9 months after diagnosis of the index case

## Other planned outcome measures are:

To compare between arms:

- The number of household’s contacts screened for TB symptoms within 3 months of treatment of the index case.
- The proportion of household contacts with unreported TB symptoms after 3 months, and the results of TB investigations initiated at the time of the 3 month home visit
- The number of household contacts diagnosed with culture confirmed TB within 3 months of treatment of the index case

In addition the acceptability of a standard of care and intervention arms to index patients, and household heads will be assessed using qualitative methods.

## Study time line

This is a 30 month study commencing in September 2012. Participant recruitment will be continued until the sample size is achieved.

## Intended use of results

This study will contribute and provide evidence base towards the current management of household contact investigation particularly in resource poor settings. Findings will help policy makers within national TB programmes. Dissemination of findings will be both local and international through regular meetings, conferences, research reviews and peer –reviewed articles with MOH collaborators and stakeholders.

# Background

Tuberculosis (TB) is caused by *Mycobacterium tuberculosis*. It is an airborne disease mainly spread through close contact with a person who has infectious TB and is coughing. The disease may present as sputum smear-positive pulmonary TB (PTB) when bacilli are visible on direct microscopy of sputum, as sputum smear-negative PTB when bacilli are not seen on direct sputum microscopy, and as extra-pulmonary TB (EPTB) when organs other than lung are affected. Sputum smear-positive PTB patients are the most infectious with the highest potential to infect other individuals in the community (2–4).National TB control programmes, therefore, earmark detection and cure of sputum smear-positive cases as the cornerstone of TB control to reduce morbidity, mortality and transmission of infection, and to prevent the development of drug resistant TB.

Malawi has a low TB case detection rate of less than 50% despite having 100% Direct Observed Treatment, Short course (DOTS) coverage (5). Although this is below the stipulated 70% case detection rate, earlier evidence has shown that globally case detection in high burden settings has remained constant at 40-50% (6,7). Recently, international policy recommendations have stressed the need to expand case finding beyond “passive case finding” (investigation of self-presenting patients complaining of TB symptoms) to improve case detection, even if DOTS coverage is good.

Screening of household contacts is one such form of “active” case finding , recommended by World Health Organization (WHO) (8–12) as illustrated in table 1 (13).

Household contact tracing can be carried out in a number of different ways, ranging from enquiry about symptoms among the household members of an index patient, with advice to bring anyone coughing to the facility, to repeated home visits and screening of all contacts for TB disease and latent TB infection. The more intensive approaches increase the yield of TB cases identified. For example, in Peru, home visits increased the detection of undiagnosed TB from 0.2% of contacts to 0.9% compared to a less intensive facility-based approach (14). Home screening of household contacts of PTB patients (“index patients”) was also shown to be effective in Malawi, but has not been widely implemented due to resource constraints (10).

In Malawi the quality of primary care TB services may contribute to low case detection. Previous studies in Malawi have reported that the poor are faced with innumerable barriers when accessing TB care due to large geographical distances and high opportunity costs of visiting health facilities (15–17)_._ In addition, shortages of drugs (such as antibiotics) used in the routine diagnostic process for smear-negative TB, and other diagnostic supplies, together with delayed recognition of TB symptoms and the inherent difficulties of confirming TB, add up to an expensive and very lengthy diagnostic experience for many TB patients (a mean of 6 visits for smear-negative TB and 4.5 visits for smear-positive reported by Kemp et al) (17).

In Malawi, and internationally, NTP activities have also failed to engage the community when compared to other programmes, such as HIV. Interventions that are based at communities have shown capability to reach the poor populations and even showing better individual outcomes (18,19). They are not necessarily more expensive if delivered by supervised community workers or by the patients themselves, and have proven to be more user friendly when compared to facility based interventions (20). In this context, considering expanding case detection by engaging communities and patients in new approaches that are aimed at detected TB cases has high potential value.

Table 1:

TB case detection rate refers to the number of reported cases per 100 000 per year divided by the estimated incidence rate per 100 000 per year

WHO case finding strategies include:

Use of “DOTS” strategy

TB screening amongst PLWHA

TB screening in Household contacts

TB screening in Prisons and congregate settings.

Source_:_ World Health Organisation.Tuberculosis care and control. A practical directory of new

advances. Map 1; Detecting more TB cases; 2011 pg 14.(13).

## Household contact tracing for TB case detection

As well as identifying active disease, contact tracing also identifies people in recent close contact with an infectious index case and, therefore, at increased risk of recent infection with *Mycobacterium tuberculosis*. About 50% of household members will be infected by sputum smear-positive index cases (12). In a recent systematic review and Meta analysis by Morrison et al, household contact tracing followed by screening identified active TB in 4.5% of contacts (2.3% with bacteriological confirmed TB). In the same meta-analysis contact screening detected a prevalence of latent TB infection with a weighted average of 51.4% (See Table 2) (12).

Consistent with these findings was a systematic review by Triasih et al that focused on children in South East Asia and found a prevalence of infection in under 15 year olds of 24%-69% with the yield of active TB disease being 3.3-5.5% (21). They also confirmed the very high risk of active TB disease in child contacts aged less than 5 years olds (21).Recently published contact investigations amongst households of index patients with multidrug resistant (MDR-TB) has also shown a high risk of both latent TB infection and active TB disease (22–24) .

Findings from Thyolo, Malawi, demonstrated that home screening of child contacts was far superior to merely inviting index patients to bring their child contacts in for assessment: the yield of TB cases was 1.7% when household screening was done as opposed to 0.2% (10) .This supports for more intensive household contact strategy in Malawi, especially for young contacts who are also a priority for IPT because of their high risk of developing the disease if infected (8,9,23) .

| *Table 2: Pooled Yield of Tuberculosis among contacts* | | | | |
| --- | --- | --- | --- | --- |
|  | Total studies | Pooled % yield (95% CI) | Heterogeneity |  |
|  |  |  | P value I^2^ |  |
| Active tuberculosis |  |  |  |  |
| Child contacts |  |  |  |  |
| < 5 year | 13 | 8.5 (7.4-9.7%) | <0.001 88.8% |  |
| 5-14 years | 6 | 6.0 (4.7-7.5%) | <0.064 43.5% |  |
| < 15 years | 8 | 7.0 (6.0-8.0%) | <0.001 88.3% |  |
| Adult contacts | 9 | 6.5 (5.7-7.4% | <0.001 70.1% |  |
| LTBI | | | | |
| Child contacts |  |  |  |  |
| < 5 year | 14 | 30.4 % (28.6-32.3%) | <0.001 94.4% |  |
| 5-14 years | 7 | 47.9% (45.5-50.4%) | <0.001 96% |  |
| < 15 years | 10 | 40.4% (38.7 – 42.2%) | <0.001 97.8% |  |
| Adult contacts | 7 | 64.6% (62.9-66.2%) | <0.001 98.7% |  |
| **From: Morrison J, Pai M, Hopewell PC. Tuberculosis and latent tuberculosis infection in close contacts of people with pulmonary tuberculosis in low-income and middle-income countries: a systematic review and meta-analysis. *Lancet Infect.Dis* 2008** | | | | |

## Contact tracing and Isoniazid Preventive Therapy (IPT)

Contact tracing and screening also identifies candidates for IPT. IPT reduces the risk of progression from recent TB infection to disease and is effective when given to recent contacts of sputum smear-positive TB cases (9,14,24,25). The effectiveness of IPT was demonstrated more than thirty years ago. It is most effective when used in those recently infected with (or exposed to) *Mycobacterium tuberculosis* (14). A recent meta- analysis of the use of IPT in HIV-negative individuals identified 11 studies with 33113 patients in which IPT versus placebo was randomly allocated. The five years cumulative incidence of TB in the placebo arm was 1.7% (range 0.4 to 34.3%),with a risk reduction of 60% (risk ratio 0.4;95% CI 0.31-0.52) when IPT was given for a period not less than 6 months to maximum of 12 months (25). Consistent with this, a 12 year follow up of contacts by Morán-Mendoza et al has also shown that without providing IPT (which acts as treatment for latent TB) the relative risk of developing TB in household contacts can be as high as 25 times (26) the background rates of disease.

In resource-limited settings, provision of IPT is burdensome for health systems and patients because it has to be given for at least 6 months. As such it is restricted to groups at highest risk of progression to TB disease: children aged 5 years or younger who are household contacts of PTB patients, and HIV-infected individuals of any age (9). Since contact tracing has been used to identify households at high risk of TB infection earlier, this may therefore enable the programme to detect both clinical and subclinical cases that may arise due to HIV infection, considering the high HIV prevalence rates seen in Malawi (27,28). Contact screening conducted among smear positive TB patients in Dar es Salaam, Tanzania, showed a similar risk of latent TB infection (62.5%) among HIV negative contacts compared to 61.6% in HIV-positive contacts (29).

## Barriers to TB diagnosis in Malawi.

Tuberculosis is among the top ten single causes of adult illness and the second most common cause of death from communicable diseases in Malawi in 2011 (30). The greatest impact of TB is on the poor, who live in crowding conditions and have poor nutrition status, putting them at increased risk of both TB infection and progression to TB disease. The poor are faced with innumerable barriers when accessing TB care due to geographical distances, financial and high opportunity costs that are involved when seeking TB diagnosis (15–17,31,32). In Malawi TB is mainly HIV-related, and lay knowledge of the relationship between HIV and TB can also act as a deterrent to seeking TB diagnosis (32)._._

Barriers to uptake of preventive interventions, such as IPT, tend to be even more pronounced than barriers to curative services. For IPT, for example, low completion rates are reported worldwide, and uptake is sub-optimal even in high income countries (33).

## Household contact policy and practice in Malawi

In Malawi contact investigation is offered to contacts of diagnosed TB patients. Household contacts of index patients are advised to come to facilities for screening. Adults who are confirmed to have active TB are placed on treatment. Six months of IPT is offered to under 5 year olds when active TB has been excluded (4)_._ However, in practice numerous barriers prevent initial investigation of contacts as there is often poor follow up or no follow up. In most cases either health workers are not aware of the rationale of conducting screening and providing IPT despite being advocated by NTP, or contacts do not report to facilities(35).

Although home-based contact tracing and screening has a higher yield of cases (10,35,36), in practice this is rarely implemented due to the high costs to the programme. In Thyolo, only 17% of eligible children came forward for TB screening at facilities. Similarly, in Blantyre District, only 9% of 365 eligible under 5 year old contacts were screened in 2011, and only 23 (6%) received IPT.

Moreover, only 21% of TB patients who had child contacts aged less than 5years below were informed about the need for screening their children (36). Nyirenda et al reported that attendance among contacts was only 7.7% (35). These findings demonstrate clear need to find an alternative approach to contact investigation and IPT that is less burdensome for the families of TB patients and yet affordable by the NTP.

We hypothesise that the low uptake relates in part to the high investment of transport costs and time that TB patients and their families need to commit in order to attend and complete the screening process. Any contact who is identified at the initial visit as symptomatic may have to make several more visits before TB is confirmed or excluded, with this being especially difficult among young children who are unable to expectorate sputum and who tend in any case to have smear-negative TB disease. Many index cases will themselves have been through a lengthy and costly diagnostic process, and so will be aware of the need for multiple visits (17).

## TB infection and disease in children

TB infection carries a relatively high risk of rapid progression to active TB that is highly age-dependent and peaks in the first year following infection or re-infection. Infection within first 5 years of life has a high risk of progression to disease (11). This is consistent with recent studies. For children less than five years old, the risk of developing TB disease in those who are infected is as high as 20-50 %, while the lifetime risk of TB disease in older children falls to about 5% (11).Therefore, incidence of cases reduces as age increase. With regards to contacts, evidence shows that children have high frequency of direct contact with index cases (70%) and particularly in resource poor settings (37,38). For these reasons IPT, which has proven to be efficacious, is targeted at those aged less than 5 year olds so as to prevent them from developing active disease (8,14,25).

Earlier studies conducted in Malawi have reported that TB infection among child household contacts is above 50% while 23- 25 % of contacts who develop TB disease will be children (10,36).More recent studies are consistent with these findings. Despite strong evidence that close contact with TB poses a great danger to children (9,11), implementation of policy is poor (39). In part this relates to the difficulties of confirming or excluding TB infection and disease in young children. In Malawi, availability of chest radiography (required to investigate suspected TB in young children and smear-negative adult TB suspects) is limited to a few facilities per district. This, therefore, poses as a challenge to implementing contact screening, as child contacts who have TB symptoms cannot be investigated at primary care level.

# Study Rationale

# National TB control programme policy is that contact tracing should be carried out at facility level. However, this has lead to poor attendance from contacts, and low uptake of IPT. Our intention is to use a cluster randomized design to investigate whether or not an alternative approach delivered primarily through the index patient/parent or guardian of child contacts will provide a more effective and potentially sustainable service. To our knowledge this is the first such study to be investigated, in Malawi and elsewhere.

# Research Question

This study intends to answer the following main question:

“Can contact tracing and screening be provided in a way that is effective and sustainable to TB patients and families in resource poor settings?”

Research Hypothesis

- that standard models of delivering contact screening, which are facility based, are ineffective in part because of the high burden of transport costs and time that are placed on TB patients and their families-that the greater participation and yield of TB cases shown in studies visiting contacts at home will be sustained in interventions delivered through index case patients themselves.

- that a Patient/parent-delivered contact screening model that aims to minimize the cost and inconvenience to the family will provide substantially higher uptake with TB symptom screening, higher yield of cases, and higher uptake and completion rates for IPT (if eligible)

# Research Aims and Objectives

The broad aim of this study is to pilot, implement and rigorously assess the effectiveness of a home based TB screening intervention to the households of TB patients in urban Blantyre, aiming to identify a potentially sustainable model that could improve uptake with all components of the current contact screening policy of NTP Malawi.

# Specific Objectives

1. To investigate the cumulative incidence of timely (within 3 months) diagnosis and treatment of TB in household contacts of adult index TB patients recruited from QECH, using a cluster randomized trial design to compare the PACTS intervention against standard of care (SOC)
2. To investigate the uptake of IPT by 3 months in under 5 year old child contacts of adult index TB patients recruited from QECH, comparing results between the two trial arms.
3. To investigate the proportion of child contacts in the two trial arms who both initiate *and* complete a 6 month course of IPT
4. To investigate the prevalence of undiagnosed TB by TB screening at a home visit conducted 3 months after the diagnosis of the index case

For objectives 1 to 3, initiation of TB treatment and IPT will be considered up to the time of (but not including) a planned 3 month household visit.

# Methodology

## Trial Design

This will be a cluster randomized trial of approximately 428 index PTB patients, providing an estimated 1712 household contacts. Index patients will be randomly allocated to the intervention or standard of care arms using simple, constrained, randomization in a 1:1 ratio

## Flow diagram

**Analysis of Outcome Measures**

Diagnosis of active TB in all household contacts

Retention and completion of IPT amongst under 5 years old

Parent/Patient Based contact tracing and screening (PACTS).

Facility Based contact tracing and screening (Standard of care).

**Patient Follow up**

3 month after randomization has occurred, and 6 months after treatment completion.

Eligible PTB patients

**Randomize**

Household Contact tracing and Screening of PTB patients at QECH Blantyre Malawi: Cluster Randomized Trial.

## Setting and Study population

This study will be conducted at QECH hospital, in Blantyre, Malawi. This facility is a central hospital and acts as a referral hospital. This centre contributes 83% of diagnosed TB cases in Blantyre district alone.

**Inclusion criteria**

- PTB new patients registering at QECH
- age 18 years or older for both male and female
- self-report of one or more under 5 year old household contact
- residence within Blantyre city boundary
- able and willing to provide written consent to participate in the study, including a 3 month household visit.
- if an inpatient: likely to be discharged within a two week period

Contacts will be defined as members of the same household (defined as sharing meals) or other residents of the same dwelling who share physical space such as sleeping or living rooms. Participation by contacts will be through oral consent, using a patient information leaflet.

**Exclusion criteria**

- unable or unwilling to provide informed consent
- hospitalized patients who are unlikely to be discharged within a two week period (e.g. due to retreatment requiring on TB treatment, and if transferred in and out of QECH).
- membership of a household that has already been recruited into the study

## Intervention-

Parent/Patient/Household Based contact screening:

The trial will investigate a newly-developed approach of: -

1. Initiating contact screening at home by the patient themselves, with the index patient responsible for: -
   1. Triaging household members who need TB investigation, or are eligible for IPT, and those where no further action is indicated
   2. Collecting and bringing sputum for examination from symptomatic household members who are otherwise well
   3. Initiating other actions as indicated, aided by “contact referral cards” that can be used to directly access paediatric services for under 5 year old family members with symptoms of TB
2. The index patient can then bring all children eligible for IPT with them to their next routine appointment (TB drugs are collected weekly from primary care clinics), where IPT can be initiated. If the index patient is not the parent/guardian, then the child(ren) will have to be accompanied by their parent/guardian
3. Thereafter the index patient, or parent of the children (if not the index patient) can assume responsibility for collecting isoniazid and carrying out monthly symptom screening for isoniazid side-effects on behalf of all family members, thereby minimizing the need for travel

The screening tool will include questions about cough, fever, weight loss, night sweats, and (for young children) failure to thrive.

## Standard of Care Arm:

Facility Based contact screening:

1. In this arm household contacts will receive the routine screening provided by the recruiting facility (QECH). Facility guideline are to:
2. Advise discharged PTB patients that household contacts (sharing meals and sleeping together) must report to the facility to be screened for TB.
3. Contacts who attend are then questioned for TB symptoms. Children are examined and the parent/guardian is asked about TB symptoms such as failure to thrive
4. All adult and children contacts who are found to be coughing or who have signs of TB submit sputum, and /or have chest radiography. If TB is confirmed, treatment is commenced.
5. Children under five years old, who are well, are put on IPT for 6 months irrespective of HIV status.
6. Parents to household contacts under 5 years old on IPT are offered education on adherence and drug safety
7. All household contact suspected of TB are registered in the TB contact register. All children offered IPT, are registered in the IPT register.IPT is given on monthly basis with the child attending the facility along with the parents.
8. HIV testing and counseling is offered to all contacts, with referral for HIV care(including IPT if indicated) if found to be HIV infected

## Sample size

The sample size of 428 clusters uses the following assumptions for the first primary outcome:

- between -cluster coefficient of variation, k = 0.30
- a cumulative incidence of 2% of active TB detected in contacts under the control arm within 3 months of diagnosis of the index case
- a cumulative incidence of 5% of active TB detected in contacts under the intervention arm within 3 months of diagnosis of the index case

Power is set as 80%. We assume 20 % of contacts will be lost to follow up at 3 months, or will have refused the intervention.

## Randomization

Random allocation will be carried out by two data managers of two separate projects using pre-generated block randomization codes. Once a participant has consented to participate, the recruiting officer will telephone either of the data managers for the allocation arm.

Field assistants will be blinded to the trial arm when conducting home visits, and investigator blinding will be maintained.

## Cohort Follow up and 3 month home visit

A list of all household contacts (age names, gender) will be collected from each index case at the time of recruitment. Contacts will be followed up at home three months after initial registration of the index case, to provide home-based screening for undiagnosed TB and to capture cases where TB treatment or IPT have been initiated. Index cases will be told to expect a household visit three months after recruitment and given the provisional date and time.

1. Residential address will be identified using “map book” system validated for Blantyre urban by Dr Corbett’s team (40) that allows location to be precisely identified through printed colour satellite maps annotated with local landmarks
2. Locator information will be collected (up to 3 telephone numbers) at the time of recruitment
3. Index cases will be reminded of the visit by short message service (SMS) or telephone call one week beforehand
4. Visits will be conducted by trained research assistants who will be responsible for interviewing all household members and taking sputum specimens and referring patients if symptomatic.

At the 3 month home visit

1. Symptom screening will be carried out in all consenting household members (interview of guardians in the case of young children) followed by clinical or bacteriogical confirmation using sputum microscopy and culture if symptomatic, or (in the case of under 5 year olds or smear-negative adults) referral for CXR.
2. Contacts with active TB will be referred for treatment, as will any child/children under-5 year olds not already receiving IPT (and not on TB treatment: See Annex table 5 below).
3. Contacts on IPT will be followed up on monthly basis, either through home-based screening for symptom (intervention arm) or facility visits (SOC arm).
4. Cohort analysis will be carried out for all contacts started on IPT using facility registers without reference to the trial arm. Data extractor for all study contacts in the IPT registers will have a study ID, participant ID or barcode for easy identification and linking.

# Outcome evaluation

The analysis of primary and secondary outcomes will use combined study data, and routine facility data. In both cases, a quality management system with supervisor-initiated quality checks on a random sample (5% to 10% of the data set collected at that point) will be used to verify the accuracy of source data especially in the registers. We will also randomly check data collected by the research assistants.

## Capture of trial outcomes

## Active TB cases

1. Cases of active TB may be diagnosed through
2. routine “passive” case-finding at facility if patient comes in sick with symptoms of TB
3. routine or PACTS household contact screening in the first 3 months after TB diagnosis is made of the index case.
4. At the 3 month household screen, active case finding will be used in all households (SOC and PACTS) with cases found through smear, culture and referral of symptomatic (later not included in primary outcome).
5. At the time of the 3 month household screen, all contacts will be asked if they are on TB treatment and if so how and when they were diagnosed. If any household member has died household head will provide details for this member (age, sex and month of death occurred).

Active TB will be confirmed by chest x ray, or bacteriogically confirmed by sputum smear examination or by culture results, or by decision to treat made by a routine service provider (see case definition).

The primary outcome will use decision to treat as the definition of active TB (since more rigorous definitions may introduce ascertain bias between the two arms). However, since TB culture and microscopy is being implemented routinely in Blantyre under a separate study, we anticipate a reasonably high proportion will be found to have bacteriologically confirmed TB.

For undiagnosed TB cases identified through the 3 month household visit, definite and possible TB cases will be included in the analysis.

## IPT initiation and completion

1. IPT can be initiated in eligible children aged less than five years through routine or PACTS household contact screening in the first 3 months after TB diagnosis is made of the index case

Data will be collected at the time of the 3 monthly household visits, and additionally at 6 months after completion of IPT treatment, using routine MOH IPT registers. .

## Data capture tools for active TB cases, and IPT initiation and completion

TB diagnosis status and treatment for all registered patients is recorded routinely by the Ministry of Health in TB laboratory register, TB treatment registers, and TB contact registers with detailed personal information. The TB treatment register also contains HIV status data.

IPT status for all registered TB patients and their households is recorded routinely by the Ministry of Health in the TB IPT registers; with personal information.

The following source documents will be used in data extraction in both arms and (confirmation of treatment initiation and initiation and completion of IPT):

- Laboratory TB registers,
- TB treatment register,
- IPT treatment register,
- Contact screening register,
- IPT treatment cards,
- DOT treatment card.

In addition, case report forms (CRF) will be created; these will include, index case baseline forms, household baseline forms, eligibility forms, 3 month household screening form, clinical assessment forms, and follow up assessment forms.

We will use barcodes identifying both the index case (cluster level ID) and the household member. In order to identify patients from TB and IPT registers, the National TB/IPT Number and date of treatment will be extracted from TB/IPT cards at the time of the 3 month household visit for all patients started on treatment. This number includes a facility code, allowing easy location of the relevant register for each case.

## Number of cluster residents registering for TB household screening care

The named focal facilitators at the clinic (the facility TB officers) and research staff from intervention and control clusters will attend monthly meetings at which used referral cards (from intervention clusters) will be collected. They will go through all information collected.

# Data management and quality assurance

## TB screening in the households

For quality assurance purposes, unannounced spot visits to households scheduled for household visits will be carried out by a supervisor. QA days will be randomly selected (5%) from all working days. As well as confirming that the household visit is taking place, a review of procedure and practice will take place to ensure that any deviations from SOP's are promptly detected and rectified.

## Data collection

Data will be collected using standardised case record forms (CRFs) that will include demographic data on the index and contacts, socioeconomic indicators based on the 1998 and 2004 Integrated Household Survey, and literacy.

Data will also be extracted from household contact and TB registers. Study-designed contact investigation forms will complement registers.

Tools will be piloted in 25 index cases and their contacts before study commencement

## Data management

Data will be collected by trained research assistants who will be recruited prior to the commencement of the trial. Data will be managed at the project offices based at HNTI in College of Medicine. Data analysis will be carried out in-house in collaboration with HIT TB study statisticians.

All data will be entered into an electronic relational database, using Optical Character Recognition (OCR) of paper forms called Optic Mark Recognition software (Cardiff Teleforms software); SOPs will be developed for validity checks before scanning, and verification of scanned records. Inconsistencies will be investigated and resolved.

Data from intervention and standard of care will be analyzed by arm only after recruitment and follow-up has been completed and the data have been verified, double entered, checked and resolved. The final analyses will then use all the combined study data.

Protocols for managing data without breach of confidentiality are in place. Access to the final data set will be limited to the Data manager (to be identified), PI (Kruger Kaswaswa) and the supervisors (Liz Corbett and Bagrey Ngwira) and the HNTI statistician. Confidentiality will be maintained for sensitive information linked to personal identifiers using password protected databases and locked cupboards. Data files will be kept in locked offices at HNTI College of Medicine during data entry, and in a locked data store room for longer term storage.

### Data collection tools, case record forms (CRF)

These are outlined in the Appendices:

### Data security

Hard copies of data and study documentation will be kept in locked offices, and long term storage will be in locked cupboards in a locked storeroom. These will be kept for five years.

Electronic copies of data will be saved in password-protected files. All data will be backed up daily by the HNTI Data Office, with offsite back up once weekly. Backup data will be stored in a locked filing cabinet away from the office by the PI. Data forms will be kept for five years.

# Statistical consideration

This trial has two primary outcomes and one secondary outcome. The first primary outcome (cumulative incidence of TB diagnosed by 3 months: yes or no) will be assessed in all household contacts, aiming for an increase in the proportion diagnosed from 2% of contacts in the SOC arm to 5% in the contacts in the intervention arm. The second primary and secondary outcomes are also cumulative incidence (proportions) being respectively, the cumulative incidence of starting IPT within 3 months if eligible ,and of starting and completing IPT if eligible, but only apply to contacts under 5 years old.

The trial has been powered around the two primary outcomes. An 80% power is adequate for a significant result at alpha of 5% if the true difference between arms is from 2% of all contacts in the SOC arm started on TB treatment within 3 months of diagnosis of the index case to 5% started on TB treatment in the Parent/Patient intervention arm. This allows for a relatively high value of *k* ( between-cluster coefficient of variation ) of 0.3 to 0.35, assumes a mean of 4 contacts per index case, and 20% loss to follow up by three months

To increase power for the secondary trial outcome, recruitment will be restricted to index cases who report at least 1 under 5 year-old household contacts. We assume conservatively that the mean number of under-5s contacts will be 1 per household, giving 214 contacts per arm. This number will provide 80% power to detect a true difference between arms in the proportion of under-5s starting IPT from the assumed 10% likelihood in the SOC arm to 20% in the intervention arm. If the uptake of IPT in the SOC arm is as high as 20% then the study has 80% power to detect a difference if the intervention arm achieves a ≥35% uptake of IPT.

## Statistical analysis

Households of index cases will be considered as clusters in this study. Baseline characteristics (household size, age, gender, socio economic status, and the smear status and HIV status of the index case) of the index cases will be collected and compared between arms, using pre-set criteria to define imbalance between arms. In the event of imbalance, an adjusted analysis will be used. Otherwise outcomes will use unadjusted odds ratios and 95% confidence intervals calculated using logistic regression and including a random-effects model to account for clustering within households. This is the recommended approach for analysis of cluster randomized trials with more than ~20 clusters per arm with proportions as the outcome measure (41).

# Ethical consideration

## Participant information and consent forms

Written (or witnessed in the case of illiterate participants and with children under 5 years) informed consent will be taken before recruitment of: -

- Individual new PTB patients diagnosed at QECH as index cases
- Individual household members being investigated for TB at the 3 months household visit

Written or witnessed informed consent will be taken from all recruited index case patients. A separate written or witnessed informed consent form a patient information sheet (with assent for older children and guardian consent for all under 18 year olds) will be used to recruit participants at the 3 month household screening visits

We will not request a waiver of written informed consent/ascent for participants starting isoniazid preventive therapy, as this is “an international standard of care” and a policy in Malawi.

**Verbal consent**

We request a waiver of written consent for recruiting household contacts as participants in the intervention arm for the home based contact screening and triage delivered by the index case. . This is because: -

- The intervention is providing screening for TB and eligibility for isoniazid preventive therapy in line with the Ministry of Health National TB Programme policy
- The potential for harm is minimal
- A requirement for written informed consent at this step would invalidate the study (since it is the index case i.e. patient who will be responsible for carrying out the TB symptom screen and triage under 5 year olds to the correct facility for IPT or TB investigations)
- A flyer will be provided for household members to read in lieu of a patient information sheet

## Ethics Committees providing review and approval

This application will be submitted to the College of Medicine Research Ethics Committee (COMREC) in Blantyre Malawi for review and approval and to the Ethics committee of London School of Hygiene and Tropical Medicine.

# Trial governance

Trial sponsor

This study is being conducted by Kruger Kaswaswa as Principal Investigator .The Malawi National TB Control programme is the trial sponsor.

## Trial Steering Committee

A Trial Steering Committee (TSC) consisting of an independent clinically experienced Chairperson (To be identified), PACTS PI (Kruger Kaswaswa), the trial statistician (HNTI assigned statistician), the trial Data Manager, one former TB patient and one Malawian Clinician will be recruited to provide independent advice and monitoring. The role of the TSC will be as defined by the MRC (UK) guidelines. Kruger Kaswaswa will be responsible for convening email discussions, conference calls, and meetings, setting the agendas, and taking and circulating minutes.

## Trial registration

This trial will be registered with ISRCTN.Our application ref is CCT-NAPN-22076.However, an ISRCTN will not be assigned until approval has been obtained by COMREC and the Ethics Committee of LSHTM.

## Data Safety Monitoring Board (DSMB)

A DSMB will be established, as specified by in the MRC UK recommendations for clinical trials .This will have 4 members (quorum for meetings defined as 3) including 2 independent statisticians and 2 clinicians. In the context of the current trial, safety concerns are minimal and no interim analysis is planned.

## Good Clinical Practice

The principles of GCP will be adhered to, and the study team will have in-house GCP training. Modified GCP guidelines suitable for community-randomized public health interventions in resource poor settings will be developed for in-house training purposes.

## Monitoring and audit

Research assistants and supervisors will be responsible for day-to-day monitoring. Project meetings attended by all field staff will be held each month, during which any problems encountered will be discussed. Refresher training will be provided if needed.

Internal audits will be conducted within 3 months of starting the intervention. These will include audit of study documentation, data storage, protocol adherence and SAE and incident reporting. A report will be circulated among TSC members and project managers.

Kruger Kaswaswa will responsible for ensuring that problem areas identified during the audits are rectified, and for circulating details of actions taken to the TSC.

The COMREC Research Governance Officer may carry out external audit of this study, but otherwise, no other external audit will be required.

# Dissemination

Results will be disseminated through quarterly meetings with, NTP, District Health Office stakeholders and staff of Queens Elizabeth Central Hospital.

Results of study will be presented at National TB research meetings, at the annual College of Medicine research forum and presented at international research meetings. Results will be prepared for publication in international peer reviewed scientific journals.

## Policy for sharing data

Data will be primarily shared with NTP stakeholders plus other scientific affiliates to COM, but we will as far as possible facilitate data sharing with any group requesting access to study data. Where appropriate, and with the proper safeguards, data will be made freely available through the College of Medicine HNTI project.

Ethical clearance will be sought before data are transferred to other groups for secondary analysis. Priority will be given to local investigators and publicly funded international investigators with data management and sharing policies in line with those of the National TB Programme, the College of Medicine- HNTI project.

## Dissemination to communities

Results will be disseminated to communities through district TB management teams, and will be presented at district, national and international meetings, and documented and disseminated through the study report and policy documents.

# Possible constraints

The main anticipated constraint will be if participating households cannot be identified for the 3 month screen. This will be minimized through the steps outlined in Section 7.8 (Cohort Follow up and 3 month home visit).

# Budget

The total budget for this is USD 47, 997.00. This covers administrative costs start up costs, study related fees and logistic costs. These academic funds will be provided by Dr E.Corbett and Dr G.Chipungu through Helse Nord TB initiative grant. (See Annex for budget justification).

## Budget Justifications

The budge justification of this cluster randomized trial encompasses items like start up costs, salaries and wages for project staff, study support ,supplies and materials, equipment, travel, dissemination and publications, other direct costs as well as administrative costs.

## Study start up costs.

We proposed to have one motorcycle that will facilitate follow up of clusters in the district considering that participant might be coming in all areas surrounding QECH, Blantyre. We will consider giving transport fee to research assistants and follow -up study participants to places mainly considered difficult to reach. We cost transport to be at USD 120.20 per month for 10 months equivalent to $1202.00.This was based at the current transport costs. Motorcycle was estimated at $ 6010.00 from a vendor quote.

The project will be required to sort out various unforeseen administrative issues within project implementation. We shall need to provide study participants with a compensation of their time and payment of transport fees to only those that are suspected to have TB and are symptomatic in household and, are sick. We calculated based on the quote from vendor that we will require $4781.00. We will also require conducting a research training and pre testing of the tools. This is done in order to provide the necessary competencies in carrying out the project work. Based on historical costs this was estimated at $1797.00.The total cost for this component is USD 12027.00

## Salaries and wages

We will require two full time research assistant for a period of a year, four part time research assistant for a period of six months, one data manager for a one year period .The role of these persons will be data collection and follow up of household contacts in their respective clusters while the data manager will be responsible for consolidation of all data and entry. The study will also require time for a nurse and a clinician who will be in the in the clinic. The project will require 20 % of their full time in the hospital that has to be paid for with a dollar equivalent of 22.06 per day according to Malawi government rates. We will also need time for 5 TB officers whom we will only require each to give us 5 % of his time and will be paid according to the Malawi Government regulations. The total for all these costs is USD 15366.00

## Other study support direct costs

We proposed in this study to budget for other direct costs. These come in form of communication, meetings, printing of barcodes. In this we also included fuel that is required for the daily logistical activities especially follow up to those sites that are very far. We include fuel at inflation rate of 10%. We estimated the other direct cost like communications ($1860.00) and barcode printing ($500.00) based on vendor cost while the meetings were was based on historical costs ($7212.00).Total budget for this component is USD 12387.00

## Supplies and materials (Laboratory).

In this study, supplies that will be required are; sputum containers and carriers, chest x ray films, registers, and isoniazid drug. We have proposed that this study will use supplies that are routinely procured by the Malawi National TB control program. However the study will be able to pay for any cultures required (MGIT and LI) for study participants to confirm TB. We estimated that 30% (130/428) of our study cluster will require culture to confirm TB assuming that each cluster will give one participant requiring so. This was calculated based on that each test will cost $25. The total cost for this component is USD 2366.00.

## Travel

There shall be need for the principal investigator to travel and present findings to one international TB /HIV forums or conference. The probable destination is France and shall be alone. The mode of transportation is by air and will require return air tickets (USD2750) and per diems of USD 500 for five days including accommodation. Total per diem rates and accommodation will be $2500.Total travel expenses are $ 5750.00

## Dissemination and publications

We will disseminate our finding locally and through the sponsorship from the HNTI project we should be able to assist the publication and disseminate the results in Malawi.

##

Itemized Budget

# Training Provided for

This study is part of Kruger Kaswaswa PhD in TB epidemiology, focusing on household contacts of TB index patients in Blantyre at College of Medicine, University of Malawi.

# Time frame

|  | Year 2012 | | | | | | | | | | | | Year 2013 | | | | | | | | | | | | Year 2014 | | | | | | | | | | | |
| --- | --- | --- | --- | --- | --- | --- | --- | --- | --- | --- | --- | --- | --- | --- | --- | --- | --- | --- | --- | --- | --- | --- | --- | --- | --- | --- | --- | --- | --- | --- | --- | --- | --- | --- | --- | --- |
|  | 1^st^ Quarter Jan- March | | | 2^nd^ Quarter Ap - Jun | | | 3^rd^ Quarter Jul- Sep | | | 4^th^ Quarter  Oct - Dec | | | 1^st^ Quarter Jan- March | | | 2^nd^ Quarter Ap - Jun | | | 3^rd^ Quarter Jul- Sep | | | 4^th^ Quarter  Oct - Dec | | | 1^st^ Quarter Jan- March | | | 2^nd^ Quarter Ap - Jun | | | 3^rd^ Quarter Jul- Sep | | | 4^th^ Quarter  Oct - Dec | | |
| Submission of Protocol to Supervisor |  | X | X |  |  |  |  |  |  |  |  |  |  |  |  |  |  |  |  |  |  |  |  |  |  |  |  |  |  |  |  |  |  |  |  |  |
| Submission to COMREC committee |  |  |  |  | x |  |  |  |  |  |  |  |  |  |  |  |  |  |  |  |  |  |  |  |  |  |  |  |  |  |  |  |  |  |  |  |
| Expected Approval |  |  |  |  |  |  |  | x |  |  |  |  |  |  |  |  |  |  |  |  |  |  |  |  |  |  |  |  |  |  |  |  |  |  |  |  |
| Training & Piloting |  |  |  |  |  |  |  |  | x | x | x |  |  |  |  |  |  |  |  |  |  |  |  |  |  |  |  |  |  |  |  |  |  |  |  |  |
| Protocol Logistics |  |  |  |  |  |  |  |  | x | x | x | x | x | x |  |  |  |  |  |  |  |  |  |  |  |  |  |  |  |  |  |  |  |  |  |  |
| Implementation  and recruitment |  |  |  |  |  |  |  |  |  | x | x | x | x | x | x | x | x | x | x | x | x | x |  |  |  |  |  |  |  |  |  |  |  |  |  |  |
| Data collection and follow up |  |  |  |  |  |  |  |  |  |  |  | x | x | x | x | x | X | x | x | x | x | x | x | x | x |  |  |  |  |  |  |  |  |  |  |  |
| Data Analysis |  |  |  |  |  |  |  |  |  |  |  | x | x | x | x | x | x | x | x | x | x | x | x | x | x | x | x |  |  |  |  |  |  |  |  |  |
| Report Writing |  |  |  |  |  |  |  |  |  |  |  |  |  |  |  |  |  |  |  |  |  |  |  |  |  |  |  |  | x | x | x | x | x | x | x |  |
| Dissemination |  |  |  |  |  |  |  |  |  |  |  |  |  |  |  |  |  |  |  |  |  |  |  |  |  |  |  |  |  |  |  |  |  | x | x |  |
| Submission |  |  |  |  |  |  |  |  |  |  |  |  |  |  |  |  |  |  |  |  |  |  |  |  |  |  |  |  |  |  |  |  | x | x | x | x |
| Publication |  |  |  |  |  |  |  |  |  |  |  |  |  |  |  |  |  |  |  |  |  |  |  |  |  |  |  |  |  |  |  |  |  | x | x | x |

# References

1. World Health Organisation. Practical Approach to Lung Health. WHO/HTM/TB. Geneva: World Health Organisation; 2008.

2. Stop TB Partnership . Progress Report on the Global Plan to Stop Tuberculosis. Geneva: 2004.

3. World Health Organisation. Treatment of Tuberculosis: Guidelines for National Programmes. 4th ed. Geneva: 2009.

4. Ministry of health. Manual for the Malawi National Tuberculosis Control programme. 6th ed. 2010.

5. Nyirenda T. Epidemiology of Tuberculosis in Malawi. Malawi Med J. 2006;18(3):147–59.

6. Dye C, Watt CJ, Bleed DM, Williams BG. What is the limit to case detection under the DOTS strategy for tuberculosis control? Tuberculosis (Edinb.). 2003 Jan;83(1-3):35–43.

7. World Health Organisation. Global tuberculosis control ,Surveillance,Planning,Financing. World Health Organization; 2011.

8. World Health Organisation. Guidance for National TB Programmes on the Management of Tuberculosis in Children. Geneva,Switzerland: 2006.

9. World Health Organisation Stop TB Partnership Childhood TB Sub group. Chapter 4.Childhood contact screening and management. Int J Tuberc Lung Dis. 2007;11(1):12.

10. Zachariah R, Spielmann M, Harries A, Gomani P, Graham SM, Bakali E, et al. Passive versus active tuberculosis case finding and isoniazid preventive therapy among household contacts in a rural district of Malawi. Int J Tuberc Lung Dis. 2003 Nov;7(11):1033–9.

11. Rieder HL. Epidemiologic Basis of TB control. 1st ed. International Union Against Tuberculosis and Lung Disease; 1999.

12. Morrison J, Pai M, Hopewell PC. Tuberculosis and latent tuberculosis infection in close contacts of people with pulmonary tuberculosis in low-income and middle-income countries: a systematic review and meta-analysis. Lancet. 2008 Jun;8(6):359–68.

13. World Health Organisation. Tuberculosis prevention , care and control :A practical directory of new advances. Geneva 27, Switzerland .: WHO Press. WHO/HTM/TB/2011.20; 2011.

14. Hsu KH. Thirty years after isoniazid. Its impact on tuberculosis in children and adolescents. JAMA . 1984 Mar 9;251(10):1283–5.

15. Nhlema B,Kemp J,Steenbergen G,Theobald G,Tang S SB. The state of existing knowledge about TB and poverty. Int J Tuberc Lung Dis. 2003;7(supp 2):116.

16. Mauch V, Woods N, Kirubi B, Kipruto H, Sitienei J, Klinkenberg E. Assessing access barriers to tuberculosis care with the tool to Estimate Patients’ Costs: pilot results from two districts in Kenya. BMC Public Health. 2011 Jan;11(1):43.

17. Kemp JR, Mann G, Simwaka BN, Salaniponi FM, Squire SB. Can Malawi’s poor afford free tuberculosis services? Patient and household costs associated with a tuberculosis diagnosis in Lilongwe. Bull World Health Organ. 2007 Aug;85(8):580–5.

18. Corbett EL, Bandason T, Duong T, Dauya E, Makamure B, Churchyard GJ, et al. Comparison of two active case-finding strategies for community-based diagnosis of symptomatic smear-positive tuberculosis and control of infectious tuberculosis in Harare, Zimbabwe (DETECTB): a cluster-randomised trial. Lancet. 2010 Oct 9;376(9748):1244–53.

19. Zachariah, R., N., Ford, M. Philips, S., Lynch, M. Massaquoi, V. Janssens HAD. Task shifting in HIV/AIDS: opportunities, challenges and proposed actions for sub-Saharan Africa. Trans.R.Soc.Trop.Med.Hyg. 2009;(103)::549–58.

20. Jaffar, S., Amuron, B,.Foster, S., Birungi, J., Levin, J., Namara,G., Nabiryo, C., Ndembi, N,Kyomuhangi, R.,Opio, A., Bunnell, R., Tappero,J.W. , Mermin, J., Coutinho, A., GrosskurthG H. Rates of virological failure in patients treated in a home-based versus a facility-based HIV-care model in Jinja, southeast Uganda: a cluster-randomised equivalence trial. Lancet. 2009;

21. Triasih R, Rutherford M, Lestari T, Utarini A, Robertson CF, Graham SM. Contact investigation of children exposed to tuberculosis in South East Asia: a systematic review. J Trop Med. 2012 Jan;2012:301808.

22. Liu E, Cheng S, Wang X, Hu D, Zhang T, Chu C. A systematic review of the investigation and management of close contacts of tuberculosis in China. J Public Health (Oxf). 2010 Dec;32(4):461–6.

23. Rieder HL. Contacts of tuberculosis patients in high-incidence countries. Int J Tuberc Lung Dis. 2003 Dec;7(12 Suppl 3):S333–6.

24. Mosimaneotsile B, Mathoma A, Chengeta B, Nyirenda S, Agizew TB, Tedla Z, et al. Isoniazid tuberculosis preventive therapy in HIV-infected adults accessing antiretroviral therapy: a Botswana Experience, 2004-2006. J Acquire Immune defie Syndr. 2010 May 1;54(1):71–7.

25. Smieja M, Marchetti C, Cook D, Smaill F. Isoniazid for preventing tuberculosis in non-HIV infected persons ( Review ). Cochrane Database of Syst.Rev (Online). 2000;2(CD001363).

26. Morán-Mendoza O, Marion SA, Elwood K, Patrick D, FitzGerald JM. Risk factors for developing tuberculosis: a 12-year follow-up of contacts of tuberculosis cases. Int J Tuberc Lung Dis. 2010 Sep;14(9):1112–9.

27. Kwanjana, JH, Harries, AD, Gausi, F, Nyangulu, DS, Salaniponi F. TB-HIV sero prevalence in patients with Tuberculosis in Malawi. Malawi Med J. 2001;13:7–10.

28. Malawi National Statistical Office, ICF Macro Calverton Maryland USA. Malawi Demographic Health Survey Report. 2010.

29. Kifai EJ, Bakari M. Mantoux skin test reactivity among household contacts of HIV-infected and HIV un-infected patients with sputum smear positive TB in Dar es Salaam, Tanzania. East Afri. J Public Health. 2009 Aug;6(2):211–8.

30. Bowie PC. Burden of Disease Estimates for 2011 and the potential effects of the Essential Health Package on Malawi ’ s health burden. Health (San Francisco). 2011;:1–6.

31. Mann G, Squire B, Nhlema, B, Luhanga, T, Salaniponi F. Expanding DOTS? Cost effective diagnostic strategies for the poorest Malawi. In: IV World TB conference. 2002.

32. Storla DG, Yimer S, Bjune GA. A systematic review of delay in the diagnosis and treatment of tuberculosis. BMC Public Health. 2008 Jan;8:15.

33. Menzies D, Al Jahdali H, Al Otaibi B. Recent developments in treatment of latent tuberculosis infection. Indian J Med Res. 2011 Mar;133(3):257–66.

34. WHO. Treatment of Tuberculosis.Guidelines for National Programmes. 2nd ed. Geneva: World Health Organisation; 1997.

35. Nyirenda M, Sinfield R, Haves S, Molyneux EM, Graham SM. Poor attendance at a child TB contact clinic in Malawi. Int J Tuberc Lung Dis. 2006 May;10(5):585–7.

36. Claessens NJM, Gausi FF, Meijnen S, Weismuller MM, Salaniponi FM, Harries AD. High frequency of tuberculosis in households of index TB patients. Int J Tuberc Lung Dis. 2002 Mar;6(3):266–9.

37. Kruk, A., Gie, RP, Schaaf, HS MB. Symptom based screening of Child Tuberculosis contacts: Improved feasibility in resource limited setting. Pediatrics 121 : 2008;121:e1646–52.

38. Uddin, S.E, Ejaz, K, Lone, S, Raza SJ. Investiment in paediatric TB prevention in Pakistan,Loss or gain??. J Park.Med .Assoc. 2010;60:897-901:897–901.

39. Fox GJ, Dobler CC, Marks GB. Active case finding in contacts of people with tuberculosis. Cochrane Database Syst.Rev. 2011 Jan;9:CD008477.

40. MacPherson P, Choko AT, Webb EL, Thindwa D, Squire SB, Sambakunsi R, van Oesterhout J, Chunda T, Chavula K, Makombe SD, Lalloo DG CE. Development and validation of a GPS-based “map book” system for categorizing cluster residency status of community members living in high-density urban slums in Blantyre, Malawi. In press Am J Epidemiol.

41. Hayes, R, Moulton L. Cluster Randomised Trials. 1st ed. CRC Press,Taylor & Francis Group; 2009.

# Annexes

## Study suggested pathway for children

Eligible children: all children housed under PTB case

Children 0-60 Months,> 60 months

Parent conducts screening

Asymptomatic

Symptomatic

Cough/fever* >14 days or Neonate (≤30 days)

HC

(Confirm asymptomatic)

QECH Paeds TB clinic or A & E

PITC

6/12 H if:

1. Under 60 months *or*

2. Over 60 months and HIV positive and not on HAART

HC= Health Centre, A & E = Accident and Emergency, PITC = Provider Initiated Testing and Counseling

*Defined as: Coughing for two weeks with or without fever or Any serious form of illness accompanied with symptoms of TB (Weight loss, failure to thrive, night sweats) or poor weight gain.

## Study definitions

| Symptoms for TB will be defined as any of | 1. Cough of any duration, night sweats, fever, weight loss. |
| --- | --- |
| Active TB disease will be defined as | 1. bacteriogically confirmed (by sputum smear examination) or by culture results or 2. Clinically/ radiological disease with decision to treat for TB. |
| Household contact | A contact shall be defined as someone close to the index case who by location sleep together or share meals together and are sharing the air for prolonged periods of time |
| Contact screening | Initial identification of household contacts with TB and, investigation of those contacts either by clinical physical examination, or symptomatic screening or sputum smear examination. |
| Active case finding | Strategy for actively detecting disease in a defined population which includes:   - Interviewing index cases to determine high risk contacts - Interviewing identified contacts to screen for symptoms of disease and risk factors for developing disease - Initial investigation of contacts for active disease including microbiological(sputum smear and/or culture),radiological(chest x ray - Periodic follow up of close contacts over time to identify evidence of active disease and - Treatment of patients identified to have active disease during screening. |
| ***Note: our study has included all the active case finding strategies at each point as study steps.*** | |

## IPT provision

### Isoniazid preventive therapy

The provision of IPT will relate to the second primary outcome and the secondary outcome. This will be delivered through routine TB care services targeting less than 5 years old, as recommended by National TB Programme. Apart from detecting microbiological confirmed TB among contacts patients, our research question also focuses on whether or not uptake and completion of IPT can be improved when a parent/patient based service delivery is offered as opposed to the facility based service delivery.

MOH tools (TB IPT registers and IPT treatment cards) will be used to capture the outcomes within 9 months.

.

### Expected number of patients

All participants will be screened either at facility level or by parent or patient at home. These shall include all the contacts irrespective of sex that are residing with the index case at the time of study and are under 5 years old.

We assume that 10% under 5 years old are initiated and complete IPT in the SOC arm. We anticipate up to 142 under 5 year old children contacts in the 428 clusters assuming total population is 1712 household members. We anticipate initiating up to 35% of these on IPT, with completion rate of 80 % of those initiated in the intervention arm.

.

### Inclusion criteria for IPT initiation (MOH)

- All Children aged 5 years or below (0- 60 months)
- A contact to an index case who is classified to have new pulmonary TB (either smear positive or smear negative)
- Resides within intervention and standard arm cluster
- Irrespective of HIV status

### Exclusion criteria (absolute contra-indications): -

- With absolute contra-indications of isoniazid (epilepsy, psychotic illness, known liver disease, previous drug-induced hepatitis with isoniazid or during TB treatment)
- Being treated for TB
- Under investigation for suspected TB

### Dose and duration of IPT

IPT will be given once a day before breakfast for all participants at a dose of 10mg/kg body weight. The duration will be 6 months. Participants who interrupt treatment for any reason will still be considered to have completed their course provided that 6 months of drug in total is taken within a 9 month period.

### Safety and monitoring during IPT

A symptom screening specific to IPT will be done monthly during collection of IPT according to NTP guidelines.. This will be carried out by the routine TB services in the SOC arm, and by trained parents/guardians in the intervention arm.

### Hepatitis

IPT is known to be safe and well tolerated. There is a small risk of hepatitis from isoniazid, and parents will be advised to discontinue tablets and contact their, project clinical team, or paediatric clinic staff if they develop any of

- - Persistent nausea
  - Vomiting
  - Dark urine or yellow discoloration of the whites of the eyes (jaundice)

Parent/Patient or household head will receive education on how to recognize hepatitis and to immediately stop isoniazid and refer any patient who develops these symptoms or signs.

Participants with suspected liver disease will have their IPT stopped and will be referred to the Paediatric Clinic staff for review at Queen Elizabeth Central Hospital. Blood will be taken for liver function tests (sent to the COM laboratory), and a clinical review appointment at Queen Elizabeth Central Hospital paediatric clinic. If hepatitis is confirmed isoniazid hypersensitivity will be recorded in their IPT Card and IPT register, and the patient will be warned of the danger of similar reactions with IPT or TB treatment in future.

### Peripheral neuropathy

There is a risk of peripheral neuropathy in patients taking isoniazid. This risk is increased in patients who have previously been treated for TB, or are malnourished but is rare in children. Parents will be advised to observe and report any painful sensations in the fingers and toes that a child may show or express to Project TB clinical team, or at paediatric clinic immediately.

### Treatment of established isoniazid neuropathy

Peripheral neuropathy will be treated with pyridoxine. Treatment dose will be 25mg/kg. Indications to prescribe treatment will depend on severity of symptoms. These will either be:

- new onset of symptomatic peripheral neuropathy of any severity or
- Self-reported or identified at the monthly symptom screen.

Mild neuropathy that does not disturb sleep, walking, or fine motor coordination of the hands (e.g. writing and dressing), or cause undue distress will be managed with continuation of IPT plus pyrodoxine.

More severe cases interfering with sleep, causing distress or interference with daily life activities will be managed by

- discontinuation of IPT and
- 2 months of treatment with pyridoxine at the recommended treatment dose.

### Dispensing

We will dispense isoniazid procured by the National programme for this purpose. Approval will be sought from the NTP programme manager. Isoniazid (100 mg tablets single tablets) will be stored at Blantyre DHO pharmacy and QECH pharmacy, with a buffer supply held in the main TB care clinics.

The clinic IPT registers will be used to forecast requirements of IPT. The PI, District TB officer and Pharmacy Technicians will be responsible for liaison and good communication with the focal TB persons at the clinic, to ensure constant availability of supplies.

The distribution of drugs to the main TB care clinic will follow the routine 3 monthly supply done by the District TB office, and will extract numbers starting IPT from the facility IPT register.

### Adherence

IPT adherence will be monitored through monthly prescription refill records and the DOT cards given to the parent or patient. Adhering to the full course of IPT does not have the same public health implications as for TB treatment (the less is taken the less effective it is as preventive therapy).

At the end of treatment the study IPT register will be completed as

- - Treatment completed without interruption
  - Treatment completed with interruption (of up to 3 months total)
  - Treatment discontinued for medical indication
    - TB diagnosed
    - Peripheral neuropathy
    - Hepatitis
    - Other adverse event
  - Default
  - Died
  - Transferred out

### Recording and reporting adverse events

Adverse events will be captured onto patient IPT treatment cards, categorized into

- Peripheral neuropathy
- Hepatitis
- Fitting
- Other

A severity grading will be carried out for peripheral neuropathy by the routine clinic staff (see grading under pyridoxine section above)

-Mild

-Moderate or severe

Hepatitis will be recorded as “suspected” by parent or guardian onto the card and then routinely reported to clinical staff. Management of suspected hepatitis will then be taken over by Project clinical staff. The final assessment will be captured onto the IPT register, patient handheld record card, and in a Hepatitis Suspects log kept by PI.

## TB case notification in Blantyre 2010

|  | **QECH** | **BLANTYRE** | **% AT QECH** |
| --- | --- | --- | --- |
|  |  |  |  |
| **PTB** | 1946 | 2359 | 82 |
| ***Smear positive*** | 941 | 1160 | 81 |
| ***Smear negative*** | 1005 | 1199 | 84 |
| **RELAPSE** | 139 | 153 | 91 |
| **EPTB** | 745 | 860 | 87 |
| **OTHER** | 175 | 204 | 86 |
| **TOTAL** | 3005 | 3590 | 83 |

## Sample size calculations for primary outcome 1

The sample size was calculated based on a power of 80%, 95% significance level and coefficient of variation of 0·30. Based on binary end points, we calculated the number of clusters required per group based on this formula:

C =1+ (Z _α/2_+Z_β_)^2^ * [π_0_(1-π_0_)/*m* + π_1_(1-π_1_)/*m*+ *k*^2^(π_0_^2^+π_1_^2^)]/ (π_1_-π_2_) ^2^ (41)

C = number of cluster

*Z* _α/2_ =level of significance of 95 % (1.96)

*z*_β_ =power of the study of 80 % ( 0.8416)

*π_1_* = proportion in the intervention = 0.05

*π_0_* =proportion in the control = 0.02

*k=* between -cluster coefficient of variation = 0.30

*m* = number of individuals/cluster size = 4

We calculated the number of clusters to be 428. Lastly we allocated 217 clusters equally to the control groups and intervention (41).

## Training

We will recruit 6 project assistants who will be trained in introduction to the study and its objectives, data collection tools, data quality, project intervention and standard arm, research ethics and ethical approvals, project SOPs, and financial management.

The training shall be conducted before the recruitment of participants to the study. One week long course will be conducted .This shall be followed by piloting of the tools to 25 participants to facilitate refining of the tools.

We have planned to conduct the training at the site, QECH when the recruitment of project staff is completed.

The training materials will be developed by Principal investigator. A complete training package of all the documents will be distributed to participants. This will also include GCP principles.

## Household Sputum Collection Procedures:

In the intervention wing the Parent or Guardian will be required to collect sputum to all suspects that are coughing. The specimens will be collected early in the morning two days prior to the next appointment day

Before collecting sputum:

- Explain to the family member the reason for collecting sputum
- Parent or guardian will demonstrate how to cough
- Demonstrate how to open and close sputum containers

How to collect sputum samples

- Ask the contact to cough deeply into the containers with early morning specimen within two consecutive days
- Ensure that no one is standing in front of the patient producing sputum
- Avoid contamination the outside of the sputum containers with sputum,(if outside is contaminated, repeat collection with a new container)
- The volume of the sputum containers should be 3 to 5 mls

After collecting sputum specimen

- - Parent/Patient will make sure that the contact has finally closed the container
  - Contact washes hand with clean water or can use soap where possible
  - Sputum is stored in a cool place and dark place in the home
  - The two specimen are delivered during the next appointment day at the clinic using the referral card

## Ethical Approval and consent Forms

**Information sheet and Consent Forms: Recruitment of Index cases**

**Title: Providing sustainable household contact tracing and screening in TB patients and families in Blantyre Malawi (PACTS).**

**Principal Investigator: Kruger Kaswaswa**

Background

My name is…………………………………………………….., and on behalf of Kruger Kaswaswa and colleagues from college of Medicine and Malawi National TB Control Programme, are conducting a research study on controlling Tuberculosis (TB) in Blantyre District. TB is a serious illness that is spread through the air when a person with TB coughs. Sharing a house with a TB patient is known to put people at risk of catching the infection. Not everyone is infected with TB will get sick, but the risk of sickness is highest for young children especially those less than 5 years and people living with HIV and AIDS.

The aim of this study is we want to know whether or not a home based contact screening is able to make a difference to improve screening and diagnosis of TB cases in Blantyre

**Request for cooperation**

We are requesting you to be in this research study. Before you accept, we will give you information that will help you decide whether or not you to participate in the study. We will read the information to you. You may ask questions about the purpose of the study, the possible risks, and the benefits from the study, your rights as a volunteer, or anything that is not clear. When all questions have been answered you can decide whether you want to participate. Your participation will be entirely voluntary.

**What will be involved if you accept to participate?**

You have been found to have TB; your family should be screened for symptoms of TB according to national policy. If a member has symptoms then they will be investigated for TB. Family members will be required to submit sputum or will have a chest x-ray done. If we find TB, then they will be treated for 6 months by the government. If the family member is less than 5 years or 60 months old and has no TB then we will give the child a medicine called isoniazid for six months to prevent TB. Children less than 5 years or 60 months old and are sick will also be tested for HIV, and if positive they will be referred for care.

Three months after treatment, you will be visited by project team at home to screen all of your family members for TB, and to ask about who has started TB treatment or isoniazid.

**Participation**

You will be in this study for 6 months. You will be allocated to either one side or the other (random allocation) of the trial arm by the study data manager .You will be assigned to either one arm by a simple procedure like tossing a coin that will be done by a computer. This is to give all participants an equal chance of belonging to either group.

Participants in the “standard of care” arm will have routine health service contact tracing and screening.

Participants in the PACTS arm will be trained to carry out the first contact screening steps themselves. We will give you a symptom check list card and pots to collect sputum into. Any member coughing will have to submit sputum, and may have to go for a chest x ray if this is negative. You will be responsible for collecting the specimens and delivering them to us. Any sick child less than 5 years needs to be taken to paediatrics at QECH without delay. We will give you a referral card to help with this.

During the your next routine TB appointment (or earlier) you will need to bring all well children less than 5 years for isoniazid (IPT) initiation, as well as any sputum specimens for anyone with TB symptoms in the family. We will give you the results to take home. The IPT for the children can be collected by you along with your TB drugs. We will give you a leaflet to take home that explains all of this to your household members.

**Benefits and risks**

There is benefit in being screened for TB as people found at an early stage suffer less severe and prolonged illness. There are no risks associated with joining this study, which will not in any way affect your treatment.

**Right to refuse or withdraw**

If you choose to join the study you are free to drop out later. If you do not join the study you can still get medical treatment as you have done in the past.

**Confidentiality**

Personal information and information related to your behaviour will be kept private and will not be shared with anyone other than the research team. The project will assign a number to all your documents and case record form (CRF) and will keep this information in a secure place

**Cost and payment**

There is no cost to you for participating in this study. You will not be paid for this study but as a compensation of your time we will give you MK200.00 at this time of recruitment. We will refund transport at during your first routine appointment. After the 3 month household visit, we will refund transport to anyone who is sick and symptomatic, but only when they have reported to the TB clinic for investigations.

**Ethical consideration**

The College of Medicine research committee has approved this study. If you have any questions about your rights as a research participant, or if you think you have not been treated fairly, you may call the University of Malawi College of Medicine Research and Ethics Committee (COMREC), College of Medicine P/Bag 360, Chichiri, Blantyre 3.Telephone number 01 871 911 or 01 874 377 Ext 209.For more information, please call Mr. Kruger Kaswaswa (Tel. 0999040929) or Dr Elizabeth Corbett (Tel. 01919304 or 0999981439) or Dr James Mpunga (Tel 0999 314914 0r 0888 314 914). To leave the study, please inform Mr. Kruger Kaswaswa (Tel. 0999040929).

**Title: Providing sustainable household contact tracing and screening in TB patients and families in Blantyre Malawi (PACTS).**

Principal Investigator: Kruger Kaswaswa

**Consent for recruiting cases**

I have been read the information sheet that explains the reasons for the study, and I understand all the procedures that I am being asked to participate in if I agree to take part in the study.

All the questions I had about this study have been answered.

I understand that:

- My household will be screened for TB using questions and then a sputum test, or chest x-ray if needed, as part of my usual TB care.
- The sputum test or chest x-ray will be provided by the study and carried out in this clinic.
- My household member will be offered free TB treatment if they are found to have TB.
- My child or household member less than 5years will be offered a drug (isoniazid) to prevent TB if they do not have TB.
- My child or household member less than 5years who is sick will be offered an HIV test as part of routine diagnosis.
- I understand that I may withdraw from this study at any time without giving a reason and without affecting my normal care and management.

I voluntarily agree:

To be traced at home or work if I miss a clinic visit.

That the information collected from me can be used for future studies.

I voluntarily agree to take part in this study:

| Participants Name (*in capital letters)* | Signature / Thumb print | Date of signature |
| --- | --- | --- |
|  |  | \|___\|___\|/\|___\|___\|___\|/201\|___\|  D D M O N Y |
|  |  |  |

If illiterate, name and signature of witness:

| Witness Name (*if illiterate)*  *in capital letters* | Signature | Date of signature |
| --- | --- | --- |
|  |  | \|___\|___\|/\|___\|___\|___\|/201\|___\|  D D M O N Y |
|  |  |  |

Study Staff taking consent ID number |___|___|___|

| Name of study staff taking consent (*in capital letters)* | Signature | Date of signature |
| --- | --- | --- |
|  |  | \|___\|___\|/\|___\|___\|___\|/201\|___\|  D D M O N Y |
|  |  |  |

## Information sheet and Consent Forms: At 3 month household screening.

Title: Providing sustainable household contact tracing and screening in TB patients and families in Blantyre Malawi (PACTS).

**Principal Investigator: Kruger Kaswaswa**

Background

My name is…………………………………………………….., and on behalf of Kruger Kaswaswa and colleagues from college of Medicine and Malawi National TB Control Programme, are conducting a research study on controlling Tuberculosis (TB) in Blantyre District. TB is a serious illness that is spread through the air when a person with TB coughs. Sharing a house with a TB patient is known to put people at risk of catching the infection. Not everyone is infected with TB will get sick, but the risk of sickness is highest for young children especially those less than 5 years and people living with HIV and AIDS.

The aim of this study is we want to know whether or not a home based contact screening is able to make a difference to improve screening and diagnosis of TB cases in Blantyre

**Request for cooperation**

We are requesting you to allow us screen you and all the family members since this family has a TB patient. But before that, we will give you information that will help you decide whether or not you to participate in the study. We will read the information to you. You may ask questions about the purpose of the study, the possible risks, and the benefits from the study, your rights as a volunteer, or anything that is not clear. When all questions have been answered you can decide whether you want to participate. Your participation will be entirely voluntary.

**What will be involved in this proces**s?

I will ask you a few questions about your personal information and about tuberculosis. You are aware that because one of your members have been found to have TB; your family should be screened for TB. If you have symptoms then you will be investigated for TB. You will be requested to submit sputum or will have a chest x-ray. If we find TB then you will be treated for 6 months by the government.

If you have a child less than 5 years with no TB symptoms, we will refer your child to receive medicine called isoniazid for six months to prevent TB. If your child is less than 5 years and has symptoms you will be referred to hospital for more investigations. There your child will also be tested for HIV and, if positive will be referred for HIV care.

We will also ask if you or your child has started TB treatment or isoniazid preventive therapy. If so we will ask to see the TB or IPT treatment card given to all patients, and will want to record the date of treatment and your patient number.

**Benefits and Risks**

There is benefit in being screened for TB as people found at an early stage suffer less severe and prolonged illness. If you have any illnesses while in study, you will be referred for treatment. There are no risks associated with joining this study and will not in any way affect treatment of your relative.

**Right to refuse or withdraw**

You are free to join the study or not. If you choose to join the study you are free to withdraw at anytime. If you do not join the study you can still get medical treatment as you have done in the past. Your participation will be now up to the end of the treatment of the index case.

**Confidentiality**

Personal information and information related to your behaviour will be kept private and will not be shared with anyone other than the principal investigator. The project will assign a number to all your documents and questionnaire and will keep this information in a secure place.

**Cost and payment**

There is no cost to you for participating in this study. You will not be paid for this. However for those that will be screened and are sick, and symptomatic, we will refund their transport when they report to clinic for further investigation.

**Ethical consideration**

The College of Medicine Research committee has approved this study. If you have any questions about your rights as a research participant, or if you think you have not been treated fairly, you may call the University of Malawi College of Medicine Research and Ethics Committee.(COMREC), College of Medicine P/Bag 360, Chichiri, Blantyre 3.Telephone number 01 871 911 or 01 874 377 Ext 209.For more information, please call Mr. Kruger Kaswaswa (Tel. 0999040929), or Dr Elizabeth Corbett (Tel. 01919304 or 0999981439) or Dr James Mpunga (Tel 0999 314914 0r 0888 314 914). To leave the study, please inform Mr. Kruger Kaswaswa (Tel. 0999040929).

**Title: Providing sustainable household contact tracing and screening in TB patients and families in Blantyre Malawi (PACTS).**

Principal Investigator: Kruger Kaswaswa

**Consent forms for household screening at 3 months.**

I have been read the information sheet that explains the reasons for the study, and I understand all the procedures that I am being asked to participate in if I agree to take part in the study.

All the questions I had about this study have been answered.

I understand that:

- I will be screened for TB using questions and then a sputum test, or chest x-ray if needed, as part of TB care.
- The sputum test or chest x-ray will be provided by the study and carried out in the TB clinic.
- I will be offered free TB treatment if I am found with TB.
- If my child or any contact is less than 5years, will be offered a drug (isoniazid) to prevent TB if I do not have TB.
- If my child or any contact is less than 5 years and sick they will be offered an HIV test as part of routine diagnosis.
- I will be asked about TB treatment or IPT started in the last 3 months
- I understand that I may withdraw from this study at any time without giving a reason and without affecting my normal care and management.

I voluntarily agree:

To be traced at home or work if I miss a clinic visit.

That the information collected from me can be used for future studies.

I voluntarily agree to take part in this study:

| Participants Name (*in capital letters)* | Signature / Thumb print | Date of signature |
| --- | --- | --- |
|  |  | \|___\|___\|/\|___\|___\|___\|/201\|___\|  D D M O N Y |
|  |  |  |

If illiterate, name and signature of witness:

| Witness Name (*if illiterate)*  *in capital letters* | Signature | Date of signature |
| --- | --- | --- |
|  |  | \|___\|___\|/\|___\|___\|___\|/201\|___\|  D D M O N Y |
|  |  |  |

Study Staff taking consent ID number |___|___|___|

| Name of study staff taking consent (*in capital letters)* | Signature | Date of signature |
| --- | --- | --- |
|  |  | \|___\|___\|/\|___\|___\|___\|/201\|___\|  D D M O N Y |

**Title: Providing sustainable household contact tracing and screening in TB patients and families in Blantyre Malawi (PACTS).**

**Principal Investigator: Kruger Kaswaswa**

**Assent Forms : Household screening for children aged between 10 years and 18 years.**

I, understand that my parents/guardian have/has given permission for me to participate in a study concerning household screening and provision of IPT under the direction of Kruger Kaswaswa

My participation in this project is voluntary and I have been told that I may stop my participation in this study at any time without penalty and loss of benefit to myself.

*Guardian or parent name*

_____________________ ___________________________

Guardian’s or parent signature Date

_______________________

Child, Name

______________________ _______________________

Investigator Signature Date

## Uthenga ndi Chilolezo: Kulemba ndi kulowetsa anthu odwala matenda a TB mukafufukuyi. (At recruitment -Chichewa version)

**Mutu:Kafufuku wofuna kupeza njira zothandizila anthu omwe akhoza kukhala ndi matenda a TB, mu gulu la anthu amene akukhala ndi munthu odwala matenda a TB**

**Mkulu wakafukufuku: Kruger Kaswaswa**

**Mau oyamba a Kafukufuku**

Dzina langa ndine........................................................ndipo ndikuimilira a Kruger Kaswaswa amene ali m’modzi mwa ophunzira za umoyo pa sukulu ya ukachenjede yotchedwa College of Medicine ku Blantyre.TB ndimatenda akulu kwambiri omwe amafala popuma mpweya omwe munthu yemwe akudwala matendawa ngati wakhosomola.Matendawa titha kupasilana ngati munthu akupuma mpweya omwe uli ndi tizilombo ta TB.Kugona munyumba imodzi ndi munthu wodwala matendawa kumaika pachiopyezo kwa anthuwo poti atha kutenga matendawa mosavuta.sialiyense angatenge kachilombo koyambisa matendawa angadwale, koma nkosavuta kudwala makamaka kwa ana ndinso anthu omwe ali ndi kachirombo ka HIV kapena odwala matenda a edzi.

Cholinga chakafukufukuyu ndi kuti tiziwe ngati kuyeza matendawa pakhomo kungabwerese kusintha kofufuza anthu odwala matendawa.

**Kupempha chilolezo.**

Tikukupempani kuti mutenge nawo mbali mu kafukufukuyu wofuna kuona/kupeza anthu omwe akhoza kukhala ndi matenda a TB, mu gulu la anthu amene akukhala ndi odwala matendawa pakhomo pawo. Koma musanatero ndikukupatsani uthenga oyenera omwe ungakuthandizeni kuvomereza kapena kukana apena ayi kutenga nawo mbali pa kafuku fukuyi. Tikuwerengerani chikalata chopempha chilolezo chanu komanso tikupemphani kuti muwerenge modekha chikalata chonse chokhuza uthenga onse wa kafuku fukuyi. Ndinu ololedwa kufunsa funso lili lonse lokhuza kafuku fukuyi komanso pena pali ponse pomwe simukutha kumvetsa bwino. Ngati mwakhutira ndi mayankho athu mutha kupereka maganizo anu ngati mukufuna kapena ayi kutenga nawo

gawo pa kafuku fukuyi. Mukavomera zotenga nawo mbali pa kafuku fukuyi dziwani kuti makolo anu kapena achibale ena ali wonse omwe mumakhala nawo limodzi nawo azaziwitsidwa za kafuku fukuyi.

**Kodi zochitika ndizotani ngati mungavomereze**

Mwapezeka ndichifuwa cha TB ndipo anthu onse omwe mumakhala nawo pakhomo akuyenera kufufuzidwa za matenda a TB.Ngati munthu wapakhomo apezeka ndi zizindikiro za TB azafufuzidwa.Anthuwa azayenera kupereka makhololo kapena kujambulidwa.Akazawatsimikizila kuti ali ndi chifuwa cha TB iwo azalandila mankhwala kwa miyezi isanu ndi umodzi kuchipatala cha boma.Ngati mbajamo muli mwanawochechepera zaka zisanu ndipo alibe matenda a TB tizapereka mankwala ochedwa isoniazidi kwa miyezi isanu mdi umodzi pofuna kuteteza matenda a TB.Anawanso azalandila uphungu ndi kuyezedwa kachilombo ka HIV.Ngati titawapeza ndi kachilomboka tizapereka kuchipatala kuti athandizidwe.Pakatha miyezi itatu mulandila mankhwala anthu amukafukufukuy azayendera khomo lanu kufuna kufufuza zamatendawa.i

Mukhala mukafukufukuyi kwa miyezi isanu ndi umodzi.Mukavomereza muzaikidwa mbali imozi ya kafukufukuyi.Bambo Choko ndi Bambo Chipeta ndiwo azakuuzeni mbali yomwe muli.Muzauzidwa zambiri zambaliyo mukalowa mukafukufukuyi.Muzakhala mbali imodzi yakafukufukuyi posatila umo momwe makina a akomputa angagawire.Cholinga chogawa motere nkufuna kupereka mwai kwa wina aliyense kuti atha kukhala mbali iliyonse mosakondera.Anthu ambali imodzi azzalandila chithandizo chamasiku onse pofufuza apakhomo pamene mbali inayo azathandizidwa pozozela njila zina zomwe kafukukuyi ati akhazikise.Muzayembekezera kusatila ndondomeko za mbali yomwe muzasiyidweyo.

**Ubwino ndi Chiopyezo**

Palibe chiopyezo china chilichonse zokhuzana ndi kulowa mukafufukuyi komanso sizizakhuza ndikamwedwe kanu kamankhwala.Ubwino wolowa kafukufukuyi ndi woti ngati mungadwale matenda ena ali wonse kupatula a TB muzathandizidwa.Tizapeleka mankhwala oteteza tb kwa ana azaka zochepera zaka zisanu.Nthawi zina mankhwalawa atha kubwelesa mavuto ngakhale izi sizimachitika pafupipafipi ndipo ndizosowa.anthu amukafukufukuyi azakuphunzisani kuti kuti muzindikile izi.

Muli ndi ufulu okana kapena kulowa mu kafuku fukuyu. Ngati mwasankha kulowa mukafuku fukuyi dziwani kuti mulinso ndi ufulu ochoka nthawi ili yonse. Komanso ngati simunalowe mukafuku fukuyi dziwani kuti mutha kupitirizabe kulandira chithandizo cha mankhwala ngati mmene mumachitira kale.

**Chinsisi**.

Uthenga uli onse okhuza inu uzasungidwa mwachinsisi ndipo mkulu ofufuza kafuku fukuyi ndi yekhayo amene adzadziwe china chili chonse chokhuza inu. Komanso dziwani kuti uthenga uli wonse okhuza inu uzizadziwika ndi nambala yapadera yomwe ali yense sazaidziwa ndipo uzasungidwa pa malo abwino osafikira wina aliyense.komanso muyenera kuziwa mukalowa kafukufukuyi dziwaninso kuti achibale anu azazindikila zakafukufukuyi ndiponso azatenga nawo mbali ngati angavomereze kutero.

**Mtengo komanso malipilo.**

Dziwaninso kuti pa nthawi yomwe mukulowa kafuku fukuyi simukuyenera kulipira kena kali konse komanso kuti simuzalipidwa malipilo ena ali onse potenga nawo gawo mu kafuku fukuyi. Koma tizakupatsani ndalama zokwana MK200.00 potaya nthawi yomwe mukolowa mukafukufukuyi.Kwaiwo omwe azalowe mukafufukuyi ndipo atayenderedwa patatha miyezi itatu azabwezeredwa makobiri awo a tharansipoti pokhapokhapo ngati azapezeke kuti ali odwala ndipo akuganizilidwa kuti ali ndi chifuwa cha TB panthawi yomwe afika ndi kufufuzidwa kuchipatala.

**Malamulo okhuza kafuku fukuyi**.

Nthambi ya zofufuza fuza ya pa sukulu ya ukachenjede wa udotolo ya College of Medicine yavomereza kufuku fukuyi. Mutha kufunsa funso lili lonse ngati mukutenga nawo gawo mu kafuku fukuyi komanso ngati mukuona kuti simunathandizidwe bwino mutha kulemba kalata kapena kutchaya lamya kwa wapampando wa nthambiyi ku Ethics Committee,COMREC, College of Medicine P/Bag 360, Chichiri,Blantyre 3.Nambala ya foni ndi 01 871 911 kapena 01 874 377 . Ngati mukufuna kudziwa zambiri zambiri za kafukufukuyi muthanso kutchaya lamya kwa a Kruger Kaswaswa pa 0999040929 komanso Dr James Mpunga pa 0999314914 kapena 0888314914 kapenanso Dr Elizabeth Corbett pa 01 1919 304 kapena 0999981 439 .

Mutu:Kafufuku wofuna kupeza njira zothandizila anthu omwe akhoza kukhala ndi matenda a TB, mu gulu la anthu amene akukhala ndi munthu odwala matenda a TB

Mkulu wakafukufuku: Kruger Kaswaswa

**Chilolezo**

Ndawerengeredwa ndindomeko yonse yakafukufukuyu ndi z**ifukwa zakafukufukuyi ndipo ndamvetsesa** ndondomeko zones zomwe ndiyenera kusatila ngati ndavomereza kutenga nawo mbali pakafukufukuyi.

Mafunso onse omwe ndinali nawo pakafukufukuyi ayankhidwa.

Ndikumvetsa ndikuziwa kuti :

- Anthu a pakhomo panga azafufuzidwa za matenda a TB ndipo azayezedwa makhololo kapena kujambulidwa ngati mbali yachisamaliro chamatenda anga.
- Munthu akapezeka ndi matenda a TB azalandila mankhwala a ulere
- Kwa mwana wanga kapena aliyense yemwe ali ndi zaka zochepera zisanu azalandila mankhwala a isoniazidi pofuna kuteteza matenda a TB.
- Kwa mwana wanga kapena aliyense yemwe ali ndi zaka zochepera zisanu azalandila uphungu ndi kuyezedwa kachilombo ka HIV ngati mbali yofuna kufufuza matenda a TB.
- Ndikumvetsa ndikuziwa kuti nditha kusiya kafukufukuyi nthawi ina iliyonse opanda kuperekeka chifukwa komanso opanda kusokoneza umo ndilandilira chithandizo change.

Ndikuvomereza mwakufuna kwanga:

- kufufuzidwa kunyumba kapena kuntchito ngati sinditha kubwela kuchiptala.
- Kuti uthenga omwe ungatengedwe kwaine utha kugwirisidwa ntchito pakafukufuku wina mtsogolo muno.
- Ndikuvomereza mwakufuna kwanga kutenga nawo mbali pakafukufukuyi:

| Dzina (*mmau akuluakulu)* | Siginacha / chidindo cha chala | Tsiku |
| --- | --- | --- |
|  |  | \|___\|___\|/\|___\|___\|___\|/201\|___\|  Ttsiku mwezi chaka |
|  |  |  |

Ngati saziwa kulemba ndi kuwerenga,dzina ndi chidindo cha mboni:

| Dzina la mboni (*ngati salemba)*  *Mmau akuluakulu* | Siginacha/chidindo | Tsiku |
| --- | --- | --- |
|  |  | \|___\|___\|/\|___\|___\|___\|/201\|___\|  Ttsiku mwezi chaka |
|  |  |  |

Dzina la ofufuza mukafukufukuyi ID number |___|___|___|

| Dzina la ofufuza (*mmau akuluakulu)* | Siginacha/chidindo | Tsiku |
| --- | --- | --- |
|  |  | \|___\|___\|/\|___\|___\|___\|/201\|___\|  Ttsiku mwezi chaka |

## Uthenga ndi Chilolezo: Kufufuku wofufuza za matenda a TB pa khomo patatha miyezi itatu (At 3 month household screening -Chichewa version).

Mutu:Kafufuku wofuna kupeza njira zothandizila anthu omwe akhoza kukhala ndi matenda a TB, mu gulu la anthu amene akukhala ndi munthu odwala matenda a TB

Mkulu wakafukufuku: Kruger Kaswaswa

**Mau oyamba a Kafukufuku**

Dzina langa ndine........................................................ndipo ndikuimilira a Kruger Kaswaswa amene ali m’modzi mwa ophunzira za umoyo pa sukulu ya ukachenjede yotchedwa College of Medicine ku Blantyre.TB ndimatenda akulu kwambiri omwe amafala popuma mpweya omwe munthu yemwe akudwala matendawa ngati wakhosomola.Matendawa titha kupasilana ngati munthu akupuma mpweya omwe uli ndi tizilombo ta TB.Kugona munyumba imodzi ndi munthu wodwala matendawa kumaika pachiopyezo kwa anthuwo poti atha kutenga matendawa mosavuta.sialiyense angatenge kachilombo koyambisa matendawa angadwale, koma nkosavuta kudwala makamaka kwa ana ndinso anthu omwe ali ndi kachirombo ka HIV kapena odwala matenda a edzi.

Cholinga chakafukufukuyu ndi kuti tiziwe ngati kuyeza matendawa pakhomo kungabwerese kusintha kofufuza anthu odwala matendawa..

**Kupempha chilolezo**

Tikukupempani kuti mutenge nawo mbali mu kafukufukuyu potilola kuti tikufufuzeni ngati mukhoza kukhala ndi matenda a TB, mu gulu la anthu amene akukhala ndi odwala matendawa pakhomo pawo. Koma musanatero ndikukupatsani uthenga oyenera omwe ungakuthandizeni kuvomereza kapena kukana kutenga nawo mbali pa kafuku fukuyi. Tikuwerengerani chikalata chopempha chilolezo chanu komanso tikupemphani kuti muwerenge modekha chikalata chonse chokhuza uthenga onse wa kafuku fukuyi. Ndinu ololedwa kufunsa funso lili lonse lokhuza kafuku fukuyi komanso pena pali ponse pomwe simukutha kumvetsa bwino. Ngati mwakhutira ndi mayankho athu mutha kupereka maganizo anu ngati mukufuna kapena ayi kutenga nawo gawo pa kafuku fukuyi. Mukavomera zotenga nawo mbali pa kafuku fukuyi dziwani kuti makolo anu kapena achibale ena ali wonse omwe mumakhala nawo limodzi nawo azaziwitsidwa za kafuku fukuyi.

**Kodi zochitika ndizotani ngati mungavomereze**

Ndikufunsani mafunso okhuzana ndi inu ndi matenda a TB.Mukuziwa kuti mukhala ndi mmodzi amene anapezeka ndichifuwa cha TB ndipo anthu onse omwe mumakhala nawo pakhomo akuyenera kufufuzidwa za matenda a TB.Ngati mwapezeka ndi zizindikiro za TB muzafufuzidwa.Muzayenera kupereka makhololo kapena kujambulidwa.Akazatsimikizila kuti muli ndi TB muzalandila mankhwala kwa miyezi isanu ndi umodzi kuchipatala cha boma.

Ngati muli ndi mwana wa zaka zochepera zaka zisanu ndipo alibe matenda a TB, tizapereka mankwala ochedwa isoniazidi kwa miyezi isanu ndi umodzi pofuna kuteteza matenda a TB.Ngati muli ndi mwana wochepera zaka zisanu ndipo akudwala , mwanayo azalandilanso uphungu ndi kuyezedwa kachilombo ka HIV.Ngati titakapeza kachilomboka tizakuperekani kuchipatala kuti muthandizidwe.

**Ubwino ndi Chiopyezo**

Ubwino ndioti mufufuzidwa matenda a TB.Ubwino wolowa kafukufukuyi ndi woti ngati mungadwale matenda ena ali wonse kupatula a TB muzathandizidwa.Palibechiopyezo china chilichonse zokhuzana ndi kulowa mukafufukuyi komanso sizizakhuza kamwedwe ka kamankhwala kambale wanu.Tizapeleka mankhwala oteteza tb kwa ana azaka zochepera zaka zisanu.Nthawi zina mankhwalawa atha kubwelesa mavuto ngakhale izi sizimachitika pafupipafipi ndipo ndizosowa.anthu amukafukufukuyi azakuphunzisani kuti kuti muzindikile izi.

Muli ndi ufulu okana kapena kulowa mu kafuku fukuyu. Ngati mwasankha kulowa mukafuku fukuyi dziwani kuti mulinso ndi ufulu ochoka nthawi ili yonse. Komanso ngati simunalowe mukafuku fukuyi dziwani kuti mutha kupitirizabe kulandira chithandizo cha mankhwala ngati mmene mumachitira kale.

**Chinsisi**

Uthenga uli onse okhuza inu uzasungidwa mwachinsisi ndipo mkulu ofufuza kafuku fukuyi ndi yekhayo amene adzadziwe china chili chonse chokhuza inu. Komanso dziwani kuti uthenga uli wonse okhuza inu uzizadziwika ndi nambala yapadera yomwe ali yense sazaidziwa ndipo uzasungidwa pa malo abwino osafikira wina aliyense.

**Mtengo komanso malipilo.**

Dziwani kuti simuzalipidwa malipilo ena ali onse potenga nawo gawo mu kafuku fukuyi. Kwaiwo omwe angapezeke kuti ali odwala ndipo akuganizilidwa kuti ali ndi chifuwa cha TB panthawi yomwe tikufufuza khomo lino azabwezeredwa makobiri awo omwe agwiritsa ntchito popita kuchipatala pokhapokha ngati akudwala ndipo ali ndi zizindikiro za matendawa panthawi yomwe afika ndi kufufuzidwa kuchipatala.

**Malamulo okhuza kafuku fukuyi**.

Nthambi ya zofufuza fuza ya pa sukulu ya ukachenjede wa udotolo ya College of Medicine yavomereza kufuku fukuyi. Mutha kufunsa funso lili lonse ngati mukutenga nawo gawo mu kafuku fukuyi komanso ngati mukuona kuti simunathandizidwe bwino. Mutha kulemba kalata kapena kutchaya lamya kwa wapampando wa nthambiyi ku Ethics Committee ,COMREC, College of Medicine P/Bag 360, Chichiri,Blantyre 3.Nambala ya foni ndi 01 871 911 kapena 01 874 377 . Ngati mukufuna kudziwa zambiri zambiri za kafukufukuyi muthanso kutchaya lamya kwa a Kruger Kaswaswa pa 0999040929 komanso Dr James Mpunga pa 0999314914 kapena 0888314914 kapenanso Dr Elizabeth Corbett pa 01 1919 304 kapena 0999981 439 .

**Mutu:Kafufuku wofuna kupeza njira zothandizila anthu omwe akhoza kukhala ndi matenda a TB, mu gulu la anthu amene akukhala ndi munthu odwala matenda a TB**

**Mkulu wakafukufuku: Kruger Kaswaswa**

**Chilolezo cha kafukufuku patatha miyezi itatu kuyambila pomwe odwala anayamba kulandila mankhwala.**

Ndawerengeredwa nd**indomeko yonse yakafukufukuyu ndi zifukw**a zakafukufukuyi ndipo ndamvetsesa ndondomeko zones zomwe ndiyenera kusatila ngati ndavomereza kutenga nawo mbali pakafukufukuyi.

Mafunso onse omwe ndinali nawo pakafukufukuyi ayankhidwa.

Ndikumvetsa ndikuziwa kuti :

- Anthu a pakhomo panga azafufuzidwa za matenda a TB ndipo azayezedwa makhololo kapena kujambulidwa ngati mbali yachisamaliro chamatenda anga.
- Munthu akapezeka ndi matenda a TB azalandila mankhwala a ulere
- Kwa mwana wanga kapena aliyense yemwe ali ndi zaka zochepera zisanu azalandila mankhwala a isoniazidi pofuna kuteteza matenda a TB.
- Kwa mwana wanga kapena aliyense yemwe ali ndi zaka zochepera zisanu azalandila uphungu ndi kuyezedwa kachilombo ka HIV ngati mbali yofuna kufufuza matenda a TB.
- Ndikumvetsa ndikuziwa kuti nditha kusiya kafukufukuyi nthawi ina iliyonse opanda kuperekeka chifukwa komanso opanda kusokoneza umo ndilandilira chithandizo change.

Ndikuvomereza mwakufuna kwanga:

- - kufufuzidwa kunyumba kapena kuntchito ngati sinditha kubwela kuchiptala.
  - Kuti uthenga omwe ungatengedwe kwaine utha kugwirisidwa ntchito pakafukufuku wina mtsogolo muno.

Ndikuvomereza mwakufuna kwanga kutenga nawo mbali pakafukufukuyi:

| Dzina (*mmau akuluakulu)* | Siginacha / chidindo cha chala | Tsiku |
| --- | --- | --- |
|  |  | \|___\|___\|/\|___\|___\|___\|/201\|___\|  Ttsiku mwezi chaka |
|  |  |  |

Ngati saziwa kulemba ndi kuwerenga,dzina ndi chidindo cha mboni:

| Dzina la mboni (*ngati salemba)*  *Mmau akuluakulu* | Siginacha/chidindo | Tsiku |
| --- | --- | --- |
|  |  | \|___\|___\|/\|___\|___\|___\|/201\|___\|  Ttsiku mwezi chaka |
|  |  |  |

Dzina la ofufuza mukafukufukuyi ID number |___|___|___|

| Dzina la ofufuza (*mmau akuluakulu)* | Siginacha/chidindo | Tsiku |
| --- | --- | --- |
|  |  | \|___\|___\|/\|___\|___\|___\|/201\|___\|  Ttsiku mwezi chaka |

Mutu:Kafufuku wofuna kupeza njira zothandizila anthu omwe akhoza kukhala ndi matenda a TB, mu gulu la anthu amene akukhala ndi munthu odwala matenda a TB

**Mkulu wakafukufuku: Kruger Kaswaswa**

**Chilolezo**

Chilolezo kwa ana azaka zapakatipa pa khumi limozi ndi khumi limozi ndi zisanu ndi zitatu.

Ine, ndikumvesa kuti makolo anga kapena ondiyang’anila apereka chilolezo ndikuvomereza kuti ine nditenge nawo mbali mukafukufukuyu yemwe ali wokhuzana ndi kuyeza kufufuza matenda a TB motsogololedwa ndi a Kruger Kaswaswa

Kutenga nawo mbali mukafukufukuyu mwakufuna kwanga ndipo ndauzidwa kuti nditha kusiya nthawi ina iliyonse popanda chindapusa kapena kuluza china chili chonse pamoyo wanga.

*Dzina la kholo*

_____________________ ___________________________

Chidindo cha Kholo Date

_______________________

Dzina la mwana

______________________ _______________________ Chidindo cha wofufuza Tsiku
