# Supplementary material for: Effect of patient-delivered household contact tracing and prevention for tuberculosis: A household cluster-randomised trial in Malawi
Source: PLoS One. 2022 Sep 8;17(9):e0269219. doi: 10.1371/journal.pone.0269219 (PMC9455850; doi:10.1371/journal.pone.0269219)
Supplement: S1 Table — (DOCX) [file pone.0269219.s004.docx]

# **S1 Table . IPT initiation among under 5 -year-old household contact**

|  | Univariate logistic regression | | | Multivariate logistic regression** | | |
| --- | --- | --- | --- | --- | --- | --- |
| IPT initiation | OR | 95% CI | | OR | 95% CI | |
| Arm |  |  |  |  |  |  |
| SOC | 1 |  |  | 1 |  |  |
| PACT | 1.94 | 1.07 | 3.53 | 2.27 | 1.04 | 4.98 |
| Wealth quintiles scores of households |  |  |  |  |  |  |
| Poorest | 1 |  |  | 1 |  |  |
| Poor than average | 1.28 | 0.45 | 3.65 | 1.29 | 0.44 | 3.82 |
| Average Poor | 1.68 | 0.57 | 4.9 | 1.55 | 0.51 | 4.66 |
| Wealthier than average | 3.64 | 1.13 | 11.75 | 3.25 | 1.01 | 10.48 |
| Least Poor | 8.48 | 1.98 | 36.31 | 8.23 | 1.63 | 41.50 |
| Smear-bacteriology of index case |  |  |  |  |  |  |
| S-ve culture- ve | 1 |  |  | 1 |  |  |
| S+ve culture +ve | 1.29 | 0.58 | 2.88 | 1.66 | 0.66 | 4.14 |
| S-ve culture +ve | 1.73 | 0.66 | 4.55 | 1.90 | 0.71 | 5.11 |
| ***=Multivariate: adjusted for wealth status of index case and smear status of index case, household level record* | | | | | | |
