# Supplementary material for: Effect of patient-delivered household contact tracing and prevention for tuberculosis: A household cluster-randomised trial in Malawi
Source: PLoS One. 2022 Sep 8;17(9):e0269219. doi: 10.1371/journal.pone.0269219 (PMC9455850; doi:10.1371/journal.pone.0269219)
Supplement: Attachment — Submitted filename: _Response to Reviewers_ 08122021.docx [file pone.0269219.s005.docx]

Kruger Kaswaswa

National TB Control Programme,

P/Bag 65,

Lilongwe,

DATE: 08.12.2021

Thesis Examiners.

*Through*

The Chief Editor,

Plos-one.

**RE: MANUSCRIPT REVISIONS- “Effect of patient-delivered household contact tracing and prevention for tuberculosis: a household cluster-randomised trial in Malawi”**

I would like to thank you for the careful critique of my manuscript. Please find below my responses to each of the queries and comments.

**REVIEWIER’S QUERIES & RESPONSES**

| **REVIEWER’S Queries/Comments** | **Response** | **Pages/sections revised** |
| --- | --- | --- |
| **Editors Comments** |  |  |
| **1.** Thank you for submitting your clinical trial to PLOS ONE and for providing the name of the registry and the registration number. The information in the registry entry suggests that your trial was registered after patient recruitment began. PLOS ONE strongly encourages authors to register all trials before recruiting the first participant in a study.  As per the journal’s editorial policy, please include in the Methods section of your paper: a) your reasons for your delay in registering this study (after enrolment of participants started); b) confirmation that all related trials are registered by stating: “The authors confirm that all ongoing and related trials for this drug/intervention are registered. | We have provided the reasons for delayed registration and provided the statement for confirmation | See the track changes copy Methods section |
| **2.** We note that you have referenced "Malawi Government(GOM). July 2012-June 2013 Annual Tuberculosis Report (Unpublished Report).Lilongwe: National TB Programme,Malawi Government; 2013" and "Malawi Government(GOM). July 2013-June 2014 Annual Tuberculosis Report (Unpublished Report).Lilongwe: National TB Programme,Malawi Government; 2014.". which has currently not yet been accepted for publication. Please remove this from your References and amend this to state in the body of your manuscript: "Malawi Government(GOM). July 2012-June 2013 Annual Tuberculosis Report (Unpublished Report).Lilongwe: National TB Programme,Malawi Government; 2013" and "Malawi Government(GOM). July 2013-June 2014 Annual Tuberculosis Report (Unpublished Report).Lilongwe: National TB Programme,Malawi Government; 2014.". as detailed online in our guide for authors <http://journals.plos.org/plosone/s/submission-guidelines#loc-reference-style> | We have taken out this reference in the main text | See the track changes copy Reference section; statistical analysis |
| **3.** Please include captions for your Supporting Information files at the end of your manuscript, and update any in-text citations to match accordingly. Please see our Supporting Information guidelines for more information: <http://journals.plos.org/plosone/s/supporting-information> | We have included the captions for Supporting information | See the track changes copy under Supplementary section |

Please find attached a copy of the manuscript (with and without “Track changes”) incorporating the changes made in response to the reviewer’s queries and recommendations.

Kruger Kaswaswa,PhD (E & ID)
