# Supplementary material for: Effect of patient-delivered household contact tracing and prevention for tuberculosis: A household cluster-randomised trial in Malawi
Source: PLoS One. 2022 Sep 8;17(9):e0269219. doi: 10.1371/journal.pone.0269219 (PMC9455850; doi:10.1371/journal.pone.0269219)
Supplement: Attachment — Submitted filename: _Response to Reviewers_ 16032022.docx [file pone.0269219.s006.docx]

Kruger Kaswaswa

National TB Control Programme,

P/Bag 65,

Lilongwe,

DATE: 16.03.2022

*Through*

The Chief Editor,

Plos-one.

**RE: MANUSCRIPT REVISIONS- “Effect of patient-delivered household contact tracing and prevention for tuberculosis: a household cluster-randomised trial in Malawi”**

I would like to thank you for the careful critique of my manuscript. Please find below my responses to each of the queries and comments.

**REVIEWIER’S QUERIES & RESPONSES**

| **REVIEWER’S Queries/Comments** | **Response** | **Pages/sections revised** |
| --- | --- | --- |
| **Comments to Author** |  |  |
| 1. If the authors have adequately addressed your comments raised in a previous round of review and you feel that this manuscript is now acceptable for publication, you may indicate that here to bypass the “Comments to the Author” section, enter your conflict-of-interest statement in the “Confidential to Editor” section, and submit your "Accept" recommendation.  Reviewer #1: All comments have been addressed  Reviewer #2: (No Response)  Reviewer #3: All comments have been addressed | N/A |  |
| 2. Is the manuscript technically sound, and do the data support the conclusions?  The manuscript must describe a technically sound piece of scientific research with data that supports the conclusions. Experiments must have been conducted rigorously, with appropriate controls, replication, and sample sizes. The conclusions must be drawn appropriately based on the data presented.  Reviewer #1: Yes  Reviewer #2: Yes  Reviewer #3: (No Response)  3. Has the statistical analysis been performed appropriately and rigorously?  Reviewer #1: I Don't Know  Reviewer #2: Yes  Reviewer #3: (No Response) | N/A |  |
| 4. Have the authors made all data underlying the findings in their manuscript fully available?  The [PLOS Data policy](http://www.plosone.org/static/policies.action#sharing) requires authors to make all data underlying the findings described in their manuscript fully available without restriction, with rare exception (please refer to the Data Availability Statement in the manuscript PDF file). The data should be provided as part of the manuscript or its supporting information, or deposited to a public repository. For example, in addition to summary statistics, the data points behind means, medians and variance measures should be available. If there are restrictions on publicly sharing data—e.g. participant privacy or use of data from a third party—those must be specified.  Reviewer #1: Yes  Reviewer #2: Yes  Reviewer #3: (No Response) | N/A |  |
| 5. Is the manuscript presented in an intelligible fashion and written in standard English?  PLOS ONE does not copyedit accepted manuscripts, so the language in submitted articles must be clear, correct, and unambiguous. Any typographical or grammatical errors should be corrected at revision, so please note any specific errors here.  Reviewer #1: No  Reviewer #2: Yes  Reviewer #3: (No Response) | We have addressed the typographical and grammatical errors | See all chapters and changes made |
| **6. Review Comments to the Author** |  |  |
| Reviewer #1: I have found it difficult to review the revisions made, as there is no file with track changes or other details of the revisions. Also I'm constrained doing the review as I'm currently on extended leave overseas and working from a tablet not computer in a guest house.  I still feel this paper should be published, but with minor revisions, as I've noticed another problem. The secondary outcome descriptions, i and iv (from line 208) are not clearly different Furthermore the presentation of these secondary outcomes in the results text and tables 2 and 3 don't seem to match in their order and description. Please review and edit for clarifty and consistency | We have provided a track changed copy and an accepted copy for this review.  Indeed, we agree with the reviewer that secondary outcome i and iv provides a slight difference. However, the objective was to split the undiagnosed TB between those that were reported and those identified as a result of assessment  **Secondary outcome 1** states that:  ….. *the proportion of household contacts not taking TB treatment at baseline with microbiologically-confirmed TB diagnosed between recruitment of the index case and the three month outcome assessment*    The first secondary outcome intends to establish the total number of TB contacts with bacteriogically confirmed TB soon after diagnosing index case **and** as a result of contact screening three months after diagnosing the index case.  **Secondary outcome iv**  *states that …… the proportion of household contacts not taking TB treatment at baseline with microbiologically-confirmed TB at three month assessment*  The fourth secondary outcome intends to establish the number of cases detected because of screening household contacts at three months (at that point of offering the assessment). This outcome is complementary to the first secondary outcome.  We have reviewed the text result for table 2 and 3. We have managed to order the results in line with the tables of concern. |  |
| **Reviewer #2**: Most of the comments that I made previously have been adequately addressed. There are some matters still to be addressed.  1. Some improved clarity is still needed. Suggestion for the Abstract as follows. Line 42-44: Would be better written as “Between May and December, 2014, we randomised patients with TB who consented to participate in the trial to either standard of care (SOC) or intervention (PACTS) arms. Participants randomised to PACTS received ….”. Line 47: “Patients randomised to SOC were managed in accordance with national guidelines, that is, they received verbal instruction …..”. Line 50-52: “The primary outcome was the proportion of adult contacts receiving treatment for TB within 3 months of randomisation. Secondary outcomes were the proportions of child contacts under age 5 years (U5Y) who were commenced on, and completed, TPT. Data were analyzed by logistic regression with random effects to adjust for household clustering.”. 2. Introduction is much improved. 3. It would be helpful to cite the number of people who met the eligibility criteria (464) in the results text (line 267).  4. I note the response in relation to using the intervention group (PACTS) as the reference group for odds ratios. I do not agree. In essence, the reference group should be the control group. A “risk” or odds ratio that is less than one is readily interpreted as indicating that the intervention is protective, which is what you are seeking to achieve. If you insist on using the intervention group as the reference for the expression of odds ratios, you should at least make this clear in the methods and in the results and tables when you present the results. Otherwise, most people will misinterpret them.  5. In table 2 you need a footnote to show what covariates adjusted for. | We have modified and changed line sections 41-42, 47  We have modified line 50-52 by adopting the suggestions by reviewer  We have indicated the number 742 of patients that were assessed for eligibility criteria, 464 patients met eligibility criteria and 214 consented to participate. We have modified this in the text  We agree to the reviewers point. We therefore maintain that the intervention as the reference group. We have included a statement the method section to guide the reader and in tables.  We inserted a note. We adjusted for wealth status and clustering | See Method section under track changes  See track changes notes  See track changes notes under result section  See track changes notes under Table 2 |
| **Reviewer #3: (No Response)** |  |  |
| 7. PLOS authors have the option to publish the peer review history of their article ([what does this mean?](https://journals.plos.org/plosone/s/editorial-and-peer-review-process#loc-peer-review-history)). If published, this will include your full peer review and any attached files. If you choose “no”, your identity will remain anonymous but your review may still be made public. **Do you want your identity to be public for this peer review?** For information about this choice, including consent withdrawal, please see our [Privacy Policy](https://www.plos.org/privacy-policy).  Reviewer #1: No  Reviewer #2: **Yes:** Guy B Marks  Reviewer #3: No | N/A | N/A |
|  |  |  |

Please find attached a copy of the manuscript (with and without “Track changes”) incorporating the changes made in response to the reviewer’s queries and recommendations.

Kruger Kaswaswa,PhD (E & ID)
